# Reporting Gaps and Challenges in Randomized Trials of Generative AI for Medical Use: Insights From a Systematic Review

**DOI:** 10.2196/94543

**Published:** 2026-10-28

**Authors:** Jae Eun Ko, Ji Woo Hwang, Sung Min Kim, Ji Yean Kwon

**Affiliations:** 1 Department of Regulatory Science for Medical Deivce, Dongguk University, Pildong-ro 1gil 30, Jung-gu, Seoul, 04620, Republic of Korea, 82 319615616; 2 Department of Medical Device and Healthcare, Dongguk University, Seoul, Republic of Korea; 3 Department of Medical AI Engineering, College of Advanced Convergence Engineering, Dongguk University, Seoul, Republic of Korea

**Keywords:** AI, generative artificial intelligence, randomized controlled trials, CONSORT-AI, large language models, reporting adherence, Consolidated Standards of Reporting Trials–AI

## Abstract

**Background:**

Generative AI (GenAI) is being deployed rapidly in medicine, and evidence of its clinical safety and efficacy depends on transparent reporting of randomized controlled trials (RCTs). The CONSORT-AI (Consolidated Standards of Reporting Trials–AI) extension is a benchmark for AI trial reporting, but it was drafted for conventional AI, and its applicability to GenAI has not been examined.

**Objective:**

This study aimed to assess item-level adherence to the CONSORT-AI among RCTs evaluating GenAI for medical purposes and to distinguish deficiencies attributable to nonadherence from those attributable to items that do not correspond to how GenAI interventions are specified or delivered.

**Methods:**

PubMed, CENTRAL (Cochrane Central Register of Controlled Trials), and Embase were searched for peer-reviewed, English-language RCTs published from 2022 to April 3, 2026, supplemented by backward and forward citation searching, in which patients, health care professionals, or laypeople received a GenAI intervention for a medical purpose; non-GenAI, nonmedical, nonrandomized, and non-peer-reviewed reports were excluded. Two reviewers independently screened studies, extracted data, and rated CONSORT-AI items as reported, partially reported, not reported, or not applicable using signaling questions, with disagreements resolved by a third reviewer (Cohen κ=0.81 and prevalence-adjusted, bias-adjusted κ=0.84). Risk of bias was not assessed, as the target was reporting adherence. Adherence was the proportion of reports rated “reported” per item; item-level adherence was compared across 9 characteristics using Fisher exact and Freeman-Halton tests (2-sided α=.05).

**Results:**

From 16,485 retrieved records, 74 reports from 73 RCTs (24,150 participants; median 100, IQR 50.0‐247.0) were included. Large language models (LLMs) accounted for 98.6% (73/74) of interventions, with 60.8% (45/74) using GPT-family models. Item-level reporting adherence ranged from 0% (0/72) to 98.6% (73/74). High adherence occurred in general metadata and output specification items (range 59-73, 79.7%‐98.6%). Conversely, reporting was poor for input and technical execution domains, input data eligibility (3/73, 4.1%), input acquisition/selection (9/74, 12.2%), algorithm versioning (17/74, 23.0%), trial setting integration (21/74, 28.4%), human-AI interaction (25/74, 33.8%), performance error (27/74, 36.5%), and open access/code (31/74, 41.9%). Input quality handling (Item 5[iii]) was completely unreported (0/72). Only 9 of 74 (12.2%) reports explicitly cited CONSORT-AI. While citing reports showed higher compliance for interaction and data sharing, major technical gaps persisted regardless of citation.

**Conclusions:**

This review provides the first empirical audit distinguishing general nonadherence from structural mismatches where deterministic CONSORT-AI items fail to capture GenAI mechanics. Differing from traditional AI evaluations, it reveals that key reproducibility drivers—system prompts, inference settings, and access modalities—are omitted from current standards. These empirical findings highlight critical reporting gaps in medical GenAI trials and identify domains for a tailored extension to current reporting standards, although the evidence is limited by the use of nonvalidated signaling questions. Establishing GenAI-specific trial guidelines will ensure that clinical decisions, regulatory approvals, and patient safety safeguards are grounded in reproducible and trustworthy evidence.

## Introduction

Generative AI (GenAI), built on foundation models (FMs), is a transformative technology with broad applications, and the medical domain is no exception [1-4]. Unlike conventional AI systems designed for specific purposes, GenAI can perform broader and more complex tasks, offering significant potential for cost-effectiveness [5,6]. Within a few years, these systems have moved from demonstrations of performance on medical knowledge benchmarks [7] to interventions delivered directly to patients and clinicians in clinical settings; as recently as late 2024, a review of registered and published trials of large language models (LLMs) in health care identified only 5 published trials [8]. Realizing such potential in clinical practice demands rigorous validation. Randomized controlled trials (RCTs) remain the gold standard for evaluating the clinical safety and efficacy of any new intervention—a principle that applies equally to GenAI [9].

The results of RCTs serve as foundational evidence for decision-making in policy development, regulation, and clinical practice. To ensure the reliability and transparency of such studies, the SPIRIT (Standard Protocol Items: Recommendations for Interventional Trials) and the CONSORT (Consolidated Standards of Reporting Trials) guidelines specify the minimum items that must be included in the design and reporting of RCTs [10,11]. In 2020, the CONSORT-AI extension was developed to address potential sources of bias and reporting concerns specific to RCTs involving AI interventions [12]. This extension is used as a benchmark for evaluating reporting in RCTs of AI interventions. The CONSORT-AI development team noted the need to update the guidelines as technology and its applications advance.

Since the publication of CONSORT-AI, its uptake has been assessed in RCTs of AI across health care generally [13,14], in trials appearing after the guideline was issued [15], and within specific fields, including ophthalmology [16] and primary care [17]. These reviews describe a consistent pattern. Aggregate adherence appears moderate to high: median concordance across the checklist was 90% (IQR 77%‐94%) among 65 trials [15], and 65% of applicable items were adequately reported among trials in primary care [17]. However, adherence is not evenly distributed across items. This review identified deficiencies in reporting which version of the algorithm was used, how low-quality input data were handled, performance errors and their analysis, and access to the AI intervention or code. In particular, there were no reported cases of performance errors in the field of ophthalmology [16]. No study to date has provided an in-depth analysis of RCT reporting for GenAI interventions intended for medical purposes. Furthermore, these studies interpret only whether reporting was complied with. That interpretation is sound where a checklist item and the intervention are well matched; however, it does not hold where they have not been examined.

GenAI differs fundamentally from conventional AI in its characteristics and uses [18-21]. The specific differences relevant to reporting can be identified in the reporting guidelines developed for GenAI systems. Transparent reporting of a multivariable model for individual prognosis or diagnosis–LLM (TRIPOD-LLM) targets studies that develop, fine-tune, and evaluate LLMs, requiring the reporting of LLM tuning, outputs, prompt engineering, and updating [21]. Furthermore, the Minimum Information about Clinical AI Checklist for Generative Modeling Research (MI-CLAIM-GEN) was revised to reflect that generative models differ from conventional AI in terms of training, evaluation, interpretability, and reproducibility [22]. Additionally, other frameworks have been proposed to address the provision of health information through chatbots and the development and evaluation of LLMs [23,24]. However, none of these guidelines specifically target RCTs. Given the ongoing evolution and expanding application of AI, the CONSORT-AI extension should also be continuously monitored and reviewed [12].

In this context, identifying and reviewing RCTs of GenAI published to date may help determine opportunities for the broader applicability of the CONSORT-AI extension. Therefore, this systematic review aimed to assess the extent to which RCTs evaluating GenAI for medical purposes adhere to the CONSORT-AI extension checklist items. Through this assessment, we sought to identify gaps between the practical characteristics of health care GenAI interventions and the CONSORT-AI extension, thereby informing future directions for refining GenAI RCT reporting guidelines.

## Methods

### Overview

This study was conducted in accordance with the PRISMA (Preferred Reporting Items for Systematic Reviews and Meta-Analyses) guidelines [25-27] (Checklists 1 and 2), and the protocol was prospectively registered in the International Prospective Register of Systematic Reviews (PROSPERO; CRD420251034690). Methodological changes made after registration, together with their rationale and timing, are summarized in Table S1 in Multimedia Appendix 1. The changes to the eligibility criteria and the search strategy determined the composition of the final sample, and all estimates reported here derive from that sample. The remaining changes affected how adherence was analyzed.

### Eligibility Criteria

The inclusion criteria were RCTs involving patients, health care professionals, or laypeople who used GenAI for medical purposes. All randomized designs were eligible, including parallel group and crossover trials. In this study, GenAI was defined as an AI system built on pretrained FMs that produces novel content in response to inputs [28-30]. This definition does not restrict output modalities and encompasses systems that generate text, images, video, and audio [1,28,31]. LLMs represent a subcategory in which the output modality is natural language; systems specialized for clinical applications through fine-tuning, in-context learning, retrieval-augmented generation (RAG), or prompt engineering share the identical generative mechanism and were therefore included [28-30]. Conversely, systems whose outputs are strictly restricted to discriminative results—such as classification, prediction, detection, or risk scoring—even if constructed on pretrained transformer backbones, alongside deterministic, rule-based systems that return prescripted responses, are explicitly excluded [1,29-31]. A medical purpose was defined as an intervention directed at a point in the clinical pathway of an identified recipient. This encompasses interventions concerned with the prevention, screening, diagnosis, monitoring, treatment, or management of disease, injury, or disability, as well as those conveying or interpreting health information in support of such purposes, and those supporting the delivery or documentation of care within a patient encounter. The exclusion criteria were interventions that were not GenAI, interventions not intended for medical purposes, studies that did not involve human participants, studies that were not peer reviewed, conference proceedings, preprints, review articles, non-English publications, and non-RCT designs. Reports that presented only a protocol or a trial registration without results were excluded.

### Information Sources

Considering the public release and rapid growth of GenAI since 2022, we searched PubMed, CENTRAL (Cochrane Central Register of Controlled Trials), and Embase for studies published from 2022 through April 3, 2026. All 3 databases were last searched on April 3, 2026. To identify additional eligible trials, backward and forward citation searching was conducted across all initially included studies on August 12, 2026, using the citationchaser (CRAN) tool [32]. To maintain methodological and temporal consistency with the primary database search, the eligibility of identified records was restricted to studies published or made available online on or before April 3, 2026, while records published after this date were excluded.

### Search Strategy

The search used free-text terms and Boolean operators with keywords such as GenAI, LLMs, and RCTs. To identify relevant RCTs, we combined text words and MeSH for GenAI with the Cochrane Highly Sensitive Search Strategy for identifying randomized trials [25]. The detailed search strategy is provided in Table S2 in Multimedia Appendix 2. The search was performed in compliance with PRISMA-S (Preferred Reporting Items for Systematic Reviews and Meta-Analyses literature search extension) [33].

### Selection Process

After deduplication, 2 authors (JEK and JWH) independently screened titles and abstracts against the inclusion and exclusion criteria. The 2 authors independently reviewed the full texts to determine final eligibility. Discrepancies were resolved by consensus, and if consensus could not be reached, the disagreements were adjudicated in consultation with a third author (JYK).

### Data Collection Process

For the included studies, 2 authors (JEK and JWH) independently extracted data. Bibliographic information (eg, title, authors, year of publication, and journal) and study characteristics (eg, country, sample size, study design, end users of the GenAI, intended use, and outcome measures) were extracted. To identify any information not reported in the main text, published study protocols and trial registrations were also reviewed. In addition, to assess item-level adherence, item-specific signaling questions were developed based on the CONSORT-AI checklist items and the accompanying explanation and elaboration document. Rather than generating new assessment criteria, we extracted every explicit reporting requirement expressed in these source documents, typically phrased as statements such as “should specify,” “should state,” or “should describe,” and converted each requirement into a single closed-ended question answerable from the trial report. The draft questions were developed by one author (JEK) and reviewed against the source documents by a second author (JWH), with discrepancies in wording or in the mapping of a requirement to an item resolved by discussion and adjudicated by a third author (JYK) where necessary. The signaling questions of CONSORT-AI checklist items are listed in Table 1, and the data extraction forms are provided in Table S3 and Table S4 in Multimedia Appendix 3. Disagreements in the assessment of individual reports were resolved by consensus and, when necessary, adjudicated by a third author (JYK).

**Table 1.** Signaling questions of CONSORT-AI^a^ checklist items.

Section/topic and numbers	CONSORT-AI checklist	Signaling questions
Title and structured abstract		
1 a, b(ⅰ)	Indicate that the intervention involves AI/machine learning in the title and/or abstract and specify the type of model	Is it stated in the title and/or abstract that the intervention involves AI/ML^b^? Are the terms describing the model type and architecture detailed in the abstract?
1 a, b(ⅱ)	State the intended use of the AI intervention within the trial in the title and/or abstract	Is the intended use of the AI intervention stated in the title and/or abstract? Is the disease context described in the title and/or abstract?
Introduction-background and rationale		
2 a(ⅰ)	Explain the intended use of the AI intervention in the context of the clinical pathway, including its purpose and its intended users (for example, health care professionals, patients, and public)	Is the intended use of the AI intervention within the clinical pathway included? Is the intended user of the AI intervention included?
Methods-eligible criteria		
4 a(ⅰ)	State the inclusion and exclusion criteria at the level of participants	Are the participant-level inclusion and exclusion criteria stated?
4 a(ⅱ)	State the inclusion and exclusion criteria at the level of the input data	Are the minimum requirements (eg, image resolution, quality metrics, or data format) for the input data specified at the input level? Is it stated when input-data eligibility was assessed? Is it stated how the input-data eligibility was assessed? Is it stated who assessed input-data eligibility?
Methods-trial setting		
4b	Describe how the AI intervention was integrated into the trial setting, including any onsite or offsite requirements	Are the hardware and software requirements described (eg, vendor-specific device, computing hardware, cloud integration at each site)? If site-specific changes were required, is the process clearly described (eg, fine-tuning on local data)?
Method-intervention and comparator		
5(ⅰ)	State which version of the AI algorithm was used	Is the version of the AI system used stated? Is it stated whether the same version was used as in previous supporting studies? Is it stated whether the version changed during the trial? 3‐1. If the version changed, are the changes between versions described? 3‐2. If the version changed, are the rationales for the changes described? If available, is a regulatory marking reference (eg, a unique device identifier) provided?
5(ⅱ)	Describe how the input data were acquired and selected for the AI intervention	Is how the input data were acquired for the AI intervention described? Is how the input data were selected for the AI intervention described? Is how the input data were preprocessed before analysis by the AI system described?
5(ⅲ)	Describe how poor-quality or unavailable input data were assessed and handled	Is the amount of poor quality or missing data reported? Is how low-quality or missing data were identified described? Is how low-quality or missing data were handled described? Is any minimum quality standard for the input data stated? If the minimum quality was not met, is it stated where the standard was not achieved? If the minimum quality was not met, is the handling process described including any impact on the participant care pathway? If relevant, is the same information about poor quality or unavailable data reported for the control intervention? If applicable, is it stated whether this minimum quality standard differed from the prerandomization input-data eligibility criteria?
5(ⅳ)	Specify whether there was human-AI interaction in the handling of the input data and what level of expertise was required of users	Is the human-AI interaction stated? Is the human-AI interface used to handle input data described? Is the required user expertise or any user training/instructions for input data handling described?
5(ⅴ)	Specify the output of the AI intervention	Is the output of the AI intervention clearly specified?
5(ⅵ)	Explain how the AI intervention’s outputs contributed to decision-making or other elements of clinical practice	Are downstream interventions that could affect outcomes described? Are the required user expertise and any training or instructions for interpreting the outputs described? If relevant, is comparable information provided for the comparator intervention? Is any discrepancy between intended (as specified in the trial protocol) and actual decision-making reported?
Results-harms		
19	Describe results of any analysis of performance errors and how errors were identified, where applicable. If no such analysis was planned or done, justify why not	1-1. Is it described how performance errors were identified? 1-2. Are the cases of error reported? 1-3. Are the implications of any performance error analysis reported? 1-4. Are the risk-mitigation strategies defined? 2. If no such analysis was planned or done, was the reason explained?
Open science data sharing		
25	State whether and how the AI intervention and/or its code can be accessed, including any restrictions to access or reuse	Is it clearly reported whether and how the AI intervention and/or its code can be accessed or reused?

a CONSORT:AI: Consolidated Standards of Reporting Trials–AI.

b ML: machine learning.

Extracted text corresponding to the CONSORT-AI checklist was classified into 4 categories. Items were rated as “Reported” only when all underlying signaling questions were met, “Partially reported” when at least one, but not all, signaling questions were satisfied, “Not reported” when none were satisfied, and “Not applicable” when the item could not be applied. Adherence to each checklist item was calculated as the number of reports rated “Reported” divided by the number of reports in which the item was applicable. Ratings of “Not applicable” were assigned when an item was not applicable to the trial design or intervention of a trial and were excluded from the denominator rather than counted as nonadherence. Adherence was presented in tabular form as the number and proportion of trials in each rating category for every checklist item, and graphically as stacked bar charts of the rating distribution. Items were ordered throughout according to their sequence in the CONSORT-AI checklist.

To assess reliability, 2 reviewers independently classified the items, and interrater agreement on these judgments was assessed using Cohen Kappa. To account for potential prevalence paradoxes arising from highly skewed reporting distributions across items, prevalence-adjusted bias-adjusted Kappa (PABAK) values were also calculated. The resultant overall interrater agreement was almost perfect (Kappa=0.81). Item-specific percent agreements, Kappa statistics, and PABAK values are detailed in Table S5 in Multimedia Appendix 4. Concordance was summarized as the adherence rate, defined as the proportion of studies categorized as “Reported” for each CONSORT-AI checklist item.

### Synthesis Methods

To evaluate whether CONSORT-AI reporting adherence differed according to study and intervention characteristics, subgroup analyses were performed across 9 categorical variables: region (North America, Asia, and Europe), publication year (2023, 2024, 2025, and 2026), intended use (information and education, mental health, decision support, documentation, and other), intended user (clinician, laypeople, others, and not reported), AI model family (GPT family, non-GPT, multiple models, and not reported), AI deployment (off-the-shelf, customized, bespoke, not reported), use of vignettes (vignette or nonvignette), reporting of system prompts (system prompt and nonsystem prompt), and declared adherence to CONSORT-AI as a reporting guideline (declared or not declared). These characteristics were selected because they capture study-level context (region and publication year), the clinical setting of the intervention (intended use, intended user, and use of vignettes), and technical attributes of the GenAI intervention itself (model family, deployment, and system prompt), together with declared use of the guideline. For the subgroup analyses, adherence for each CONSORT-AI item was dichotomized into “Reported” versus “Not reported or Partially reported.” The reports in which the item was rated “Not applicable” were excluded from the comparison. To compare adherence rates across subgroups, exact tests were used due to small cell frequencies: the 2-sided Fisher exact test was used for comparisons between 2 groups, and its extension (the Freeman-Halton test) was used for comparisons involving 3 or more groups. Categories that were small or heterogeneous (eg, “Other” in intended use) were excluded from formal significance testing and presented with descriptive statistics. Two-sided *P*<.05 was considered significant. Subgroup comparisons were displayed as a matrix of *P* values across items and characteristics. Because item-level comparisons were repeated across the checklist items and 9 study characteristics, false-positive findings cannot be excluded. Benjamini-Hochberg false discovery rate (FDR)–adjusted q values were therefore calculated separately within each of the 9 study characteristics across the applicable items for all comparisons. To address the possibility that items comprising a greater number of signaling questions were less likely to meet the all-or-none criterion for full reporting, adherence was additionally recalculated as the mean proportion of applicable signaling questions satisfied per item as a sensitivity analysis. All statistical analyses were conducted using Python (version 3.10.19; Python Software Foundation).

## Results

### Study Selection

Study selection is presented in Figure 1 according to the PRISMA flow diagram [26]. A total of 13,059 records were identified from 3 databases. After deduplication, 11,223 records were screened. Following full-text assessment of 181 reports, 111 reports were excluded, including one report whose full text was not accessible despite retrieval attempts [34]. Consequently, a total of 74 reports [35-108] were included in this review. In addition, backward and forward citation searching yielded 3426 records, all of which were sought and assessed for eligibility. Of these, 3422 reports were excluded, resulting in 4 additional eligible reports. In total, 74 published reports representing 73 unique RCTs were included in the review. Two reports [35,36] were separate publications of the same trial and were assessed independently, as the unit of analysis was the individual report.

**Figure 1.**
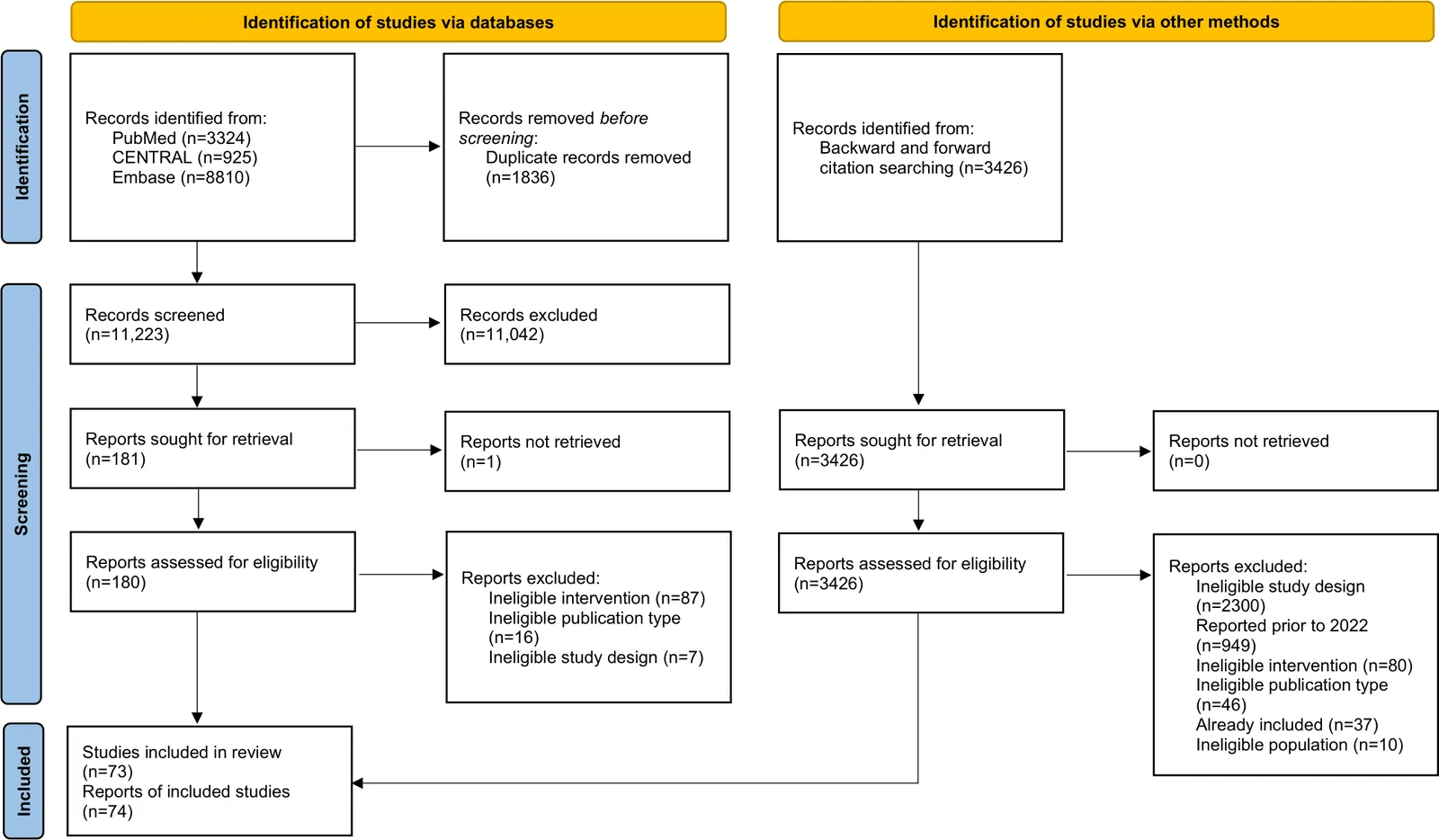
PRISMA (Preferred Reporting Items for Systematic Reviews and Meta-Analyses) flow diagram.The flow diagram outlines the identification, screening, eligibility assessment, and inclusion of trials from database searches and citation searching. A total of 74 published reports [35-108] representing 73 unique RCTs were included in this review.

### Study Characteristics

The 74 included reports (representing 73 unique RCTs) were categorized across six domains: (1) geographic region, (2) publication year, (3) primary intended use, (4) intended user, (5) AI model family, and (6) AI deployment mode. Two reports derived from the same trial were evaluated independently at the publication level. In panel (3), “Other” indicates procedural image reconstruction. In panel (5), “Non-GPT” includes non-OpenAI generative models (eg, standalone LLMs, vision-language models, and image reconstruction networks). A total of 74 reports representing 73 RCTs evaluating GenAI interventions for medical purposes were included in this systematic review, encompassing a cumulative total of 24,150 human participants (median sample size: 100 [IQR 50‐247]; range: 9‐2671). Notably, all but one [37] of the included reports used LLM-based interventions. The general and technical characteristics of the included reports are detailed in Table S6 in Multimedia Appendix 5.

Geographically, half of the reports were conducted in Asia (n=37 [35-38,40,43-45,47-49,51,54,56,58-61,63-66,68,76,77,83,84,86-89,92,93,96,102,104,105], 50.0%), followed by North America (n=23 [39,41,42,46,50,52,53,55,57,71,75,78-81,85,91,94,99,103,106-108], 31.1%) and Europe (n=14 [62,67,69,70,72-74,82,90,95,97,98,100,101], 18.9%). The publication timeline demonstrated rapid, continuous growth, beginning in 2023 (n=2 [48,49], 2.7%) and 2024 (n=8 [38-41,50-53], 10.8%), reaching a peak in 2025 (n=39 [42-47,54-81,97,99,103,107,108], 52.7%), and continuing into early 2026 (n=25 [35-37,82-96,98,100-102,104-106], 33.8%). Regarding intervention domains, the most common primary intended use was providing medical information and education to laypeople (n=33 [38,44,46,49,51,52,54,57-60,63,64,67-70,72-74,76,83,84,86,89,91-93,96,98,101,102,104], 44.6%), followed by mental health support (n=14 [35,36,47,48,61,65,66,77,78,81,82,87,100,103], 18.9%), clinical decision support (n=14 [39,42,45,53,56,62,71,75,79,80,85,90,94,105], 18.9%), and administrative support/clinical documentation (n=12 [40,41,43,50,55,88,95,97,99,106-108], 16.2%), with one report categorized as other (procedural image reconstruction). Correspondingly, laypeople and patients were the primary end users in 38 reports [35,36,38,40,44,47-49,51-54,58,59,61,63-65,69,70,73,77-79,81-84,86,87,91,93,96,100-104] (51.4%), whereas clinicians were targeted in 23 reports [39,41-43,45,50,55,56,60,62,66,71,75,80,85,92,94,95,97,105-108] (31.1%); end users were unstated or categorized as others in 13 reports [37,46,57,67,68,72,74,76,88-90,98,99] (17.6%).

Technologically, systems built upon the OpenAI GPT model family predominated (n=45 [35,36,39-41,43,47,49-54,57-60,62,64,66-75,77,78,80,81,83,86,88-90,94,95,97-99,102,105], 60.8%), followed by non-GPT (n=22 [37,42,44,45,48,55,56,65,76,79,84,85,87,91,92,100,103,104,106-109], 29.7%) and reports evaluating multiple models simultaneously (n=5 [38,63,82,96,101], 6.8%), the model family was not reported in 2 reports (2.7%). In terms of AI deployment, half of the reports relied on off-the-shelf commercial applications (n=37 [38,39,41,46,49-53,55,57,59,60,62-64,67,68,70-72,74-76,82,89,92,96-99,101,102,105-108], 50.0%), while 32 reports [35,36,40,42-45,47,54,56,58,65,66,69,73,77-81,83-88,90,91,94,95,103,104] (43.2%) used customized architectures (eg, fine-tuned or RAG-integrated systems), and 3 reports [37,48,100] (4.1%) used bespoke models; deployment mode was unreported in 2 reports [61,93] (2.7%). Regarding prompt transparency, only 20 reports [35,40,43-47,62,74,75,78-80,82,83,87,90,94,95,97] (27.0%) explicitly reported the system prompt used in the GenAI intervention. Methodologically, most reports used a parallel-group design (n=67 [35-40,42-44,46-73,75-78,80-94,96,97,100-108], 90.5%), whereas 7 reports [41,45,74,79,95,98,99] (9.5%) used a crossover design. Most reports evaluated 2 parallel/crossover arms (n=57 [35-38,40,42,44-47,49,51,52,54,56-62,64-69,71,74-81,83-86,89-91,93-95,97-100,102-108], 77.0%), while 17 reports [39,41,43,48,50,53,55,63,70,72,73,82,87,88,92,96,101] (23.0%) featured 3 or more arms. Finally, 17 reports [39,41,42,53,57,62,71,73,75,79,80,94,95,98,99,101,105] (23.0%) used clinical vignettes or standardized scenarios rather than direct real-world patient encounters. Regarding the explicit use of reporting guidelines, a total of 28 reports [35-39,43,46,50,54-56,60-62,65,66,78,80,83,85,88,89,91-93,98,102,105] (37.8%) explicitly cited adopting at least one reporting framework, with some reports following multiple guidelines. Specifically, standard CONSORT was the most frequently cited guideline (n=10 [35,36,39,54,66,78,85,92,102,105], 13.5%), followed by CONSORT-AI (n=9 [37,43,55,56,80,83,88-90], 12.2%), CONSORT-EHEALTH (Consolidated Standards of Reporting Trials of Electronic and Mobile Health Apps and Online Telehealth; n=6 [60-62,65,92,93], 8.1%) and Standards for Quality Improvement Reporting Excellence (SQUIRE; n=3 [38,46,50], 4.1%). Other cited frameworks included TRIPOD-LLM (n=1 [89]), Minimum Reporting Items for Clear Evaluation of Accuracy Reports of Large Language Models in Health Care (MI-CLEAR-LLM; n=1 [89]), Sex and Gender Equity in Research (SAGER; n=1 [37]), SPIRIT-AI (n=1 [37]), and STROBE (Strengthening the Reporting of Observational Studies in Epidemiology; n=1 [98]). Notably, most reports (n=46 [40-42,44,45,47-49,51-53,57-59,63,64,67-77,79,81,82,84,86,87,90,94-97,99-101,103,104,106-108], 62.2%) did not specify the use of any reporting guideline in their manuscript text.

### Results of Syntheses

#### Reporting Adherence of Included Studies

As shown in Table 2 and Figure 2, item-level adherence to the CONSORT-AI checklist across the 14 evaluated items varied widely among the included reports, ranging from 0% to 98.6% for full reporting. Overall, high adherence was concentrated in general trial reporting items (eg, Items 1 a, b[i], 1 a, b[ii], 2 a[i], and 4 a[i]) and output specifications (Item 5[v]), whereas partial reporting was predominant in trial implementation and technical specifications (eg, Items 4 a[ii], 4b, 5[i], and 5[ii]). Moderate-to-low adherence was observed for clinical decision contribution (Item 5[vi]: 51.5%), data sharing (Item 25: 41.9%), human-AI interaction (Item 5[iv]: 33.8%), and performance error analysis (Item 19: 36.5%). Crucially, reporting of input data quality management (Item 5[iii]) was completely absent across all applicable reports.

**Table 2.** Item-level reporting rates of the CONSORT-AI^a^ checklist.

Section/topic and number	CONSORT-AI checklist	Reporting rate (%)
Title and structured abstract		
1 a, b(ⅰ)	Indicate that the intervention involves AI/machine learning in the title and/or abstract and specify the type of model	86.49
1 a, b(ⅱ)	State the intended use of the AI intervention within the trial in the title and/or abstract	98.65
Introduction-background and rationale		
2 a(ⅰ)	Explain the intended use of the AI intervention in the context of the clinical pathway, including its purpose and its intended users (for example, healthcare professionals, patients, and the public)	79.73
Methods-eligible criteria		
4 a(ⅰ)	State the inclusion and exclusion criteria at the level of participants	89.19
4 a(ⅱ)	State the inclusion and exclusion criteria at the level of the input data	4.11
Methods-trial setting		
4b	Describe how the AI intervention was integrated into the trial setting, including any onsite or offsite requirements	28.38
Method-intervention and comparator		
5(ⅰ)	State which version of the AI algorithm was used	22.97
5(ⅱ)	Describe how the input data were acquired and selected for the AI intervention	12.16
5(ⅲ)	Describe how poor-quality or unavailable input data were assessed and handled	0.00
5(ⅳ)	Specify whether there was human–AI interaction in the handling of the input data and what level of expertise was required of users	33.78
5(ⅴ)	Specify the output of the AI intervention	94.59
5(ⅵ)	Explain how the AI intervention’s outputs contributed to decision-making or other elements of clinical practice	51.47
Results-harms		
19	Describe results of any analysis of performance errors and how errors were identified, where applicable. If no such analysis was planned or done, justify why not	36.49
Open science data sharing		
25	State whether and how the AI intervention and/or its code can be accessed, including any restrictions to access or reuse	41.89

a CONSORT-AI: Consolidated Standards of Reporting Trials–AI.

**Figure 2.**
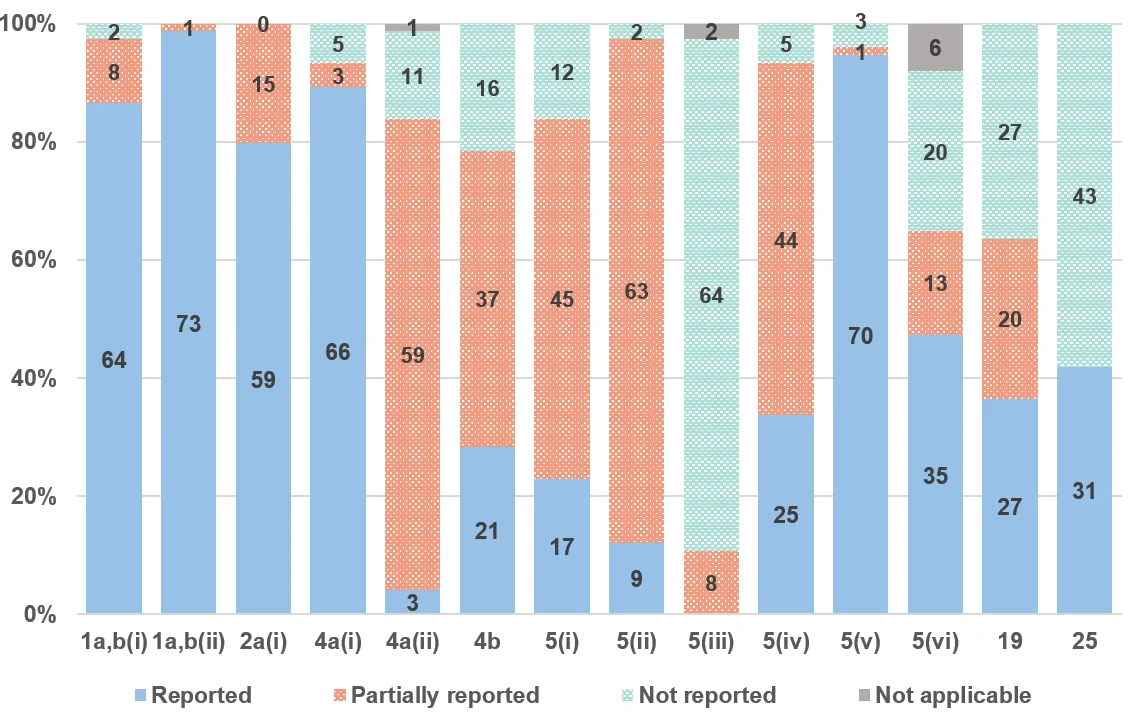
Item-level reporting status of CONSORT-AI (Consolidated Standards of Reporting Trials–AI) checklist. This stacked bar chart shows the number of studies that fully reported, not reported, partially reported, or not applicable for each CONSORT-AI checklist item. Each bar represents one checklist item, with color segments indicating reporting adherence.

Ratings of “Not applicable” accounted for 0.9% (9/1036) of the item-level observations and were confined to 3 items: Item 5(vi; n=6), Item 5(iii; n=2), and Item 4 a(ii; n=1). Denominators were therefore 74 for 11 items, 73 for Item 4 a(ii), 72 for Item 5(iii), and 68 for Item 5(vi). In a sensitivity analysis, recalculating adherence as the mean proportion of applicable signaling questions satisfied per report did not alter the overall pattern of findings, although estimates were higher for Items 5(ii; 46.2%, SD 24.4%) and 5(iv; 68.7%, SD 28.8%), indicating that partial reporting was common for these items. Item 5(iii) remained near-absent (5.3%, SD 15.9%; Table S7 in Multimedia Appendix 6).

#### Subgroup Analyses

Item-level adherence was compared across subgroups defined by region, publication year, intended use, intended user, AI model family, AI deployment, use of clinical vignettes, use of a system prompt, and whether the report cited CONSORT-AI as a reporting guideline. Item-level *P* values for all comparisons are presented in Figure 3, and group-specific proportions for each variable are provided in Tables S8-S16 in Multimedia Appendix 7. Item 5(iii) was not testable for any variable, as no article reported it.

**Figure 3.**
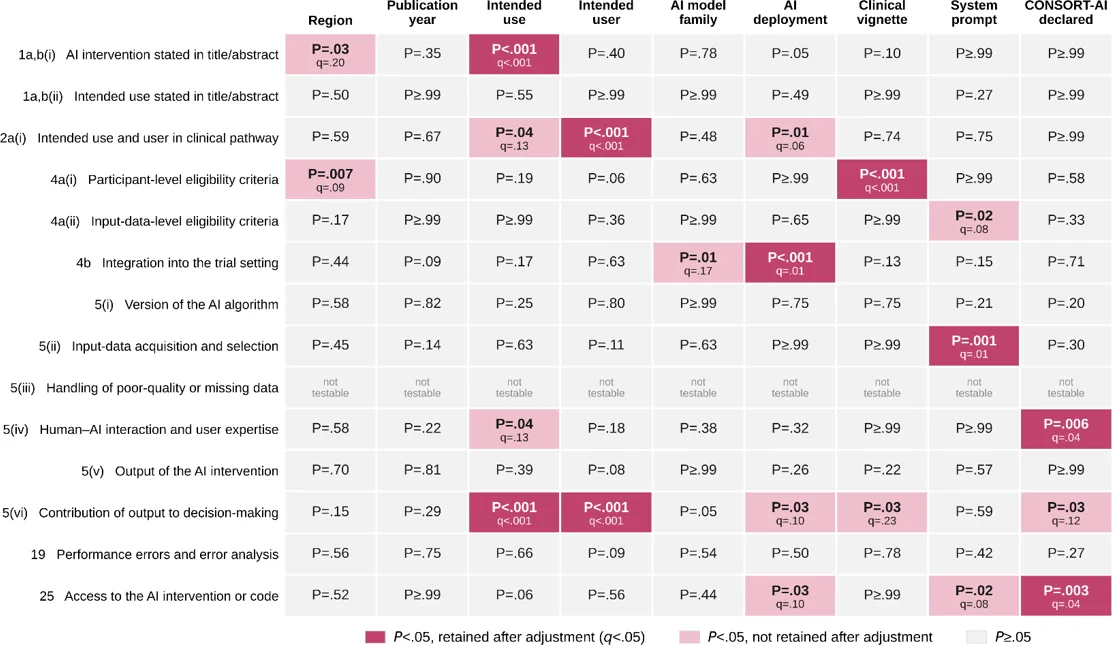
Item-level *P* values for subgroup comparisons of CONSORT-AI (Consolidated Standards of Reporting Trials–AI) adherence (n=74). Each cell shows the *P* value from an exact test comparing the proportion of reports in which the item was classified as “Reported” across groups of the corresponding variable. For cells with unadjusted *P*<.05, the FDR-adjusted q value is also displayed. Dark pink cells indicate differences retained after adjustment; light pink cells indicate differences not retained after adjustment. Item 5(iii) was not testable because no trial reported it. The group-specific proportions and Benjamini-Hochberg adjusted q values are provided in Table S8-S16 in Multimedia Appendix 7. CONSORT-AI: Consolidated Standards of Reporting Trials–AI.

The region showed differences in Items 1 a, b(i) and 4 a(i), though neither retained significance after FDR adjustment, and no Item differed by publication year. Intended use showed differences in Items 1 a, b(i), 2 a(i), and 5(vi), although only the differences in Items 1 a, b(i) and 5(vi) retained significance after adjustment, with 5(vi) separating reports in clinical decision support and clinical documentation from those in patient information and mental health. Differences by intended user were retained after adjustment for Items 2 a(i) and 5(vi). That for 5(vi) was the largest difference observed in any comparison in this review. Every clinician-facing trial reported how the intervention’s output contributed to decision-making, whereas fewer than one in 5 layperson-facing reports did so. The difference in Item 2 a(i) was definitional rather than substantive. The reports were classified as “not reported” because they did not state an intended user, as required by this item. An unadjusted difference by AI model family in Item 4b did not withstand FDR adjustment. AI deployment showed differences in Items 2 a(i), 4b, 5(vi), and 25, though only Item 4b retained significance after adjustment. The use of clinical vignettes showed a difference in Item 4 a(i), which was retained after adjustment, whereas the use of a system prompt significantly distinguished the input-related Item 5(ii). Nine reports [37,43,55,56,80,83,88-90] declared adherence to CONSORT-AI as a reporting guideline. Across 14 items, these articles reported 64.0% (80/125) of applicable items, higher than 46.6% (420/902) among the remaining 65 reports [35,36,38-42,44-54,57-79,81,82,84-87,91-108] (denominators exclude 1 and 8 item-level ratings of “Not applicable,” respectively), with differences retained after adjustment in Items 5(iv) and 25. Four items showed no difference across any of the 9 variables examined: 1 a, b(ii), 5(i), 5(v), and 19.

## Discussion

### Principal Findings

This systematic review evaluated the reporting adherence of 74 published reports representing 73 RCTs evaluating medical GenAI interventions against the CONSORT-AI checklist [12]. This discussion first examines the critical reporting gaps identified across the included trials and outlines key considerations for refining future reporting standards tailored to GenAI-based medical interventions. Our evaluation revealed substantial reporting deficiencies across critical methodological and technical domains, including complete omissions in input data quality management (5[iii]), frequent partial reporting of algorithm versioning (5[i]), and insufficient descriptions of human–AI interaction (5[iv]) and performance error analyses (19). In parallel with these widespread reporting gaps, explicit adoption of the CONSORT-AI guideline remained low, with only 9 [37,43,55,56,80,83,88-90] (12.2%) of included reports citing its use.

These observations invite different explanations. An item may go unreported because investigators were unaware that it was required, in which case the deficiency lies with adherence, and wider dissemination of the guideline is the remedy. It may go unreported because what the item asks for does not correspond to how a GenAI intervention is specified or delivered, in which case the deficiency lies with the item and the remedy is revision. Or the information needed to reproduce the report may not be requested by any item at all, in which case neither adherence nor revision suffices and the checklist must be extended.

Studies that explicitly declared adherence to CONSORT-AI demonstrated higher compliance for code/model accessibility (Item 25) and input interaction requirements (Item 5[iv]) compared to nonciting studies (*P*<.05; q<.05). While an unadjusted difference was also observed for output decision-making contribution (Item 5[vi]), this association was attenuated after FDR adjustment and represents an exploratory finding. These statistical findings suggest that reporting opacities in Items 5(iv) and 25 are attributable to investigator nonadherence, which can be mitigated by enforcing existing reporting standards.

Specifying the instructions or knowledge required for users to interpret outputs and detect potential errors is needed, yet reporting for this domain remained limited (Item 5[vi]: 51.5%). This gap is further reflected in our subgroup analysis: reports with clinicians as the intended AI users achieved a 100% (23/23) full reporting rate for Item 5(vi), whereas reports with laypeople as the intended AI users achieved a full reporting rate of 15.8% (6/38; *P*<.001). Laypeople bring no baseline expertise, and the interpretive burden that GenAI transfers to the user is correspondingly heavier [110]. Reports of laypeople-facing interventions should therefore state what instructions participants were given for interpreting outputs.

Additionally, the level of professional expertise or guidance required during human–AI interaction while handling input data (Item 5[iv]) was fully reported in only 33.8% of reports. Reporting varied across clinical application domains: it was higher in domains where clinicians are the primary intended users—such as clinical decision support (6/14, 42.9%) and administrative/clinical documentation (7/12, 58.3%)—and lower in domains where laypeople are the primary intended users, such as informational and educational support (10/33, 30.3%) and mental health support (1/14, 7.1%). Although this domain-level variation was exploratory after FDR adjustment, this pattern suggests that trials of laypeople-facing conversational tools less often report the user instructions and interaction safeguards required for operating GenAI interventions.

Beyond user interaction, reporting regarding open science principles was similarly suboptimal, with intervention and code accessibility (Item 25) fully specified in only 41.9% of reports. Importantly, without a statement of whether and how the intervention or its underlying prompts, parameters, and code can be obtained, subsequent researchers cannot access the exact intervention evaluated, let alone validate it [111]. Although subgroup differences across deployment modes did not retain statistical significance after adjustment, this low reporting was consistent with trials using commercial off-the-shelf models, where the artifact is controlled by a third party and may not persist in the form tested [112].

By contrast, explicit citation of CONSORT-AI was not associated with higher reporting adherence for the other items. This pattern suggests that while enforcing CONSORT-AI can resolve deficiencies in general interaction and accessibility items (Items 5[iv] and 25), it remains insufficient on its own to rectify reporting gaps in remaining deployment and technical specifications (such as Items 4b, 4 a[ii], 5[i], 5[ii], and 5[iii]). The remainder of this discussion therefore examines each of these items in turn—how it was drafted, why it fails for GenAI interventions, and what form it would need to take instead—before considering the conditions that determine GenAI output but that no CONSORT-AI item presently requests at all.

Descriptions of how the GenAI intervention was integrated into the trial environment, including site-specific or external requirements (Item 4b), were reported in 28.4% of reports. Given that most included interventions were LLMs or commercial API-based services, the remaining studies did not report baseline operational prerequisites, such as required internet connectivity or target access devices (eg, mobile phones or desktop computers). Subgroup analysis showed that this reporting rate varied by the deployment mode. Reports using commercial off-the-shelf models (eg, ChatGPT) demonstrated a significantly lower full reporting rate for Item 4b (5/37, 13.5%) compared to customized (12/32, 37.5%) or bespoke (3/3, 100%) interventions (*P*=.001; q=.012). Conversely, while reports using GPT family models exhibited lower reporting adherence for Item 4b than non-GPT models (*P*=.01), this difference was attenuated after adjustment (q=.173), indicating that deployment rather than AI model family drives reporting transparency. This disparity highlights the possibility that investigators using ubiquitous commercial models frequently operate under the assumption that widespread familiarity obviates the need for explicit documentation of basic connectivity and deployment prerequisites.

The 3 CONSORT-AI items concerning input data had the lowest reporting rates in this review. Input-data eligibility criteria 4 a(ii) was reported in 4.1% of reports, input-data acquisition and selection (5[ii]) were reported in 12.2%, and handling of poor quality or unavailable input data 5(iii) was reported by none. All 3 items were drafted for interventions that operate on data assembled before a trial begins, whereas, in 73 of the 74 reports, we reviewed an LLM-received text generated during the trial itself. In conventional AI systems, input data such as medical images or biosignals are structured and quantifiable, allowing researchers to assess their quality in terms of resolution, data format, and quality metrics [12]. Since these inputs can be standardized through the preprocessing procedure, Items 4 a(ii), 5(ii), and 5(iii) of the CONSORT-AI could be systematically reported. In contrast, in most GenAI systems reported here, the input is composed at the point of use, most often by the intended user, and its properties are determined by that person rather than by the protocol. The same intervention consequently receives materially different input from one participant to the next. This variation is not incidental to performance. Language models have been shown to vary in accuracy by 8%-50% according to how a query is optimized [113] and by 5%-18% according to the order in which the same information is presented [114].

Item 4 a(ii) of the CONSORT-AI checklist requires researchers to specify the minimum input requirements necessary for the AI intervention to achieve its intended use prior to randomization. This item is essential for ensuring reproducibility under consistent conditions and enhancing the reliability of result interpretation. As noted earlier, it is inherently difficult to manage inputs in clinical trials involving GenAI, which likely contributes to the low level of adherence to this reporting item. Nevertheless, it is necessary to predefine at least basic inclusion and exclusion criteria for inputs at the prompt level. Allowing users to freely generate prompts can be beneficial for assessing real-world applicability and understanding how input variation affects output quality. However, the absence of predefined input criteria undermines reproducibility [115]. Item 4 a(ii) is therefore best revised rather than removed. Where investigators structure the input—through prompt templates, standardized scenarios, or predefined instructions—eligibility can be stated at the level of that material: the language in which input is accepted, permissible length, the clinical elements it must contain for the intervention to function as intended, and the categories of content that must not be submitted. Where unconstrained interaction is intentional to assess real-world performance, that decision should be stated explicitly and its consequences acknowledged.

Item 5(ii), which asks how input data were acquired and selected for the AI intervention prior to analysis by the AI system, was fully reported in only 9 reports [43,45,46,60,62,74,78,90,99] (12.2%) and by only one [78] of the 38 reports [35,36,38,40,44,47-49,51-54,58,59,61,63-65,69,70,73,77-79,81-84,86,87,91,93,96,100-104] in which laypeople were the intended users. In our evaluation, full reporting was credited exclusively to reports that defined explicit data gating procedures. These included either structural constraints at the user interface level—such as enforcing structured input templates or predefined prompt instructions—or human-in-the-loop protocols in which clinical implementers or investigators filtered, verified, and submitted patient data into the AI system on the users’ behalf. Conversely, trials permitting unconstrained, free-text generation by interacting users were categorized as nonreporting because the tasks of data selection, structuring, and preprocessing were entirely delegated to the individual intended user without investigator oversight.

This empirical finding highlights a major methodological vulnerability in conversational GenAI trials: when user interaction is unconstrained, investigators may treat input data handling not as a controlled methodological step but as an unreportable artifact of user behavior. However, unconditioned user inputs introduce variability that directly impacts GenAI performance [38,39,116]. Even in unconstrained, free-text conversational interventions, investigators control input selection and acquisition at the backend through prompt engineering architectures, such as system prompts, or automated input-filtering rules prior to model inference [40-45]. This is directly shown by our subgroup analysis, where reporting of a system prompt was associated with higher adherence to Item 5(ii) after adjustment (*P*=.001; q=.014).

Therefore, our findings demonstrate that Item 5(ii) remains highly relevant but requires explicit contextual adaptation for GenAI. Future reporting standards should refine Item 5(ii) to mandate disclosure at 2 operational levels: first, at the user interface level, by stating whether inputs are governed by structured templates, clinician mediation, or unconstrained free text; and second, at the system architecture level, by detailing backend prompt engineering, context selection, and preinference filtering protocols executed before the AI system analyzes the query.

Item 5(iii) was reported by no trial in this review. Reviews of conventional AI trials have also found this item to be among the least reported [13,14,16,17], and to that extent the deficiency is not new. Its character in GenAI trials is nonetheless different and, in our view, more consequential. Item 5(iii) requires authors to describe how low-quality or missing input data are handled. For structured input, “poor quality” has a referent that can be defined in advance and detected automatically; for example, an image below a resolution threshold, a corrupted file, or a missing field. When user queries serve as inputs, ambiguity, inconsistency, lack of context, or omission of key terms can degrade input quality [117,118]. The difficulty compounds where the intervention involves sustained interaction. As the interaction continues, each turn generates new input that cannot be screened before it reaches the model.

In this context, unavailable data can represent inputs that should not be submitted, namely protected health information (PHI). PHI includes identifiers that can be used to recognize an individual and cover past, present, or future physical or mental health conditions [119]. In the United States, PHI is strictly regulated under the Health Insurance Portability and Accountability Act (HIPAA). Any GenAI system handling PHI must comply with HIPAA requirements like any other health care technology. However, the web-based interface of ChatGPT, the most used model in the reviewed studies, is not HIPAA-compliant [120]. This means patient information must never be entered into the public web interface. Instead, a HIPAA-eligible cloud environment that offers a Business Associate Agreement (BAA), such as the Azure OpenAI Service [121], should be used. The trials in this review were conducted in 16 countries, with 21 reports [39,41,42,46,50,53,55,57,71,75,78-81,85,91,94,99,103,107,108] from the United States. Trials conducted elsewhere are governed by their own regimes, including the General Data Protection Regulation (GDPR) in Europe [122], and what constitutes adequate protection is therefore not a single standard. What is common across them is that the answer depends on the interface through which data were submitted and the contractual arrangement under which they were processed, which is precisely what trial reports do not currently state. Eight reports addressed such matters partially and none completely.

Trial reports should state what constitutes an inadmissible input, what interface arrangement governed the data submitted, what measures prevented participants or operators from submitting inadmissible content, and what was done when this occurred. MI-CLAIM-GEN takes a comparable approach, asking not how such data were processed but what steps were taken to understand privacy and security concerns and distinguishing between discussion, identification, and mitigation of them [22].

Item 5(i) was reported in 23.0% of articles, and adherence did not differ across any of the 9 variables examined. Version reporting has been found deficient in reviews of conventional AI trial reports [13-16], but the 2 settings differ in whether the intervention stays fixed once the trial begins. A conventional model evaluated in a trial is ordinarily locked, and its identity is stable for as long as the trial runs. Off-the-shelf GenAI systems are not. They are continuously updated with new training data, model weights, and fine-tuning methods [123,124]. Many of these models are updated on short cycles and rolled out without public changelogs, making it difficult to ensure consistent study outcomes [125]. Even models under the same name, such as GPT-4, exist in multiple versions (eg, gpt-4‐0314, gpt-4‐0613, gpt-4-turbo, and gpt-4o), each with distinct performance profiles [125]. An article that reports having used “GPT-4” has partially satisfied Item 5(i) as written but has not supplied what the item exists to secure. The difficulty is not only that version labels are coarse. Item 5(i) refers to the version of “the AI algorithm,” in the singular, whereas a contemporary GenAI intervention is frequently an assembly of components. MI-CLAIM-GEN accordingly asks for the base model together with any embedding models, retrieval models, and auxiliary models or tools [22], and TRIPOD-LLM asks for the model name, version, and last date of training, and separately for the architecture, fine-tuning procedure, and alignment strategy [21]. An intervention combining a base model with retrieval over a local document set and a fixed set of instructions cannot be identified by a version string, however precise. That an item can be met without meeting its purpose is a limitation of the item rather than of the authors who complied with it, and it indicates that the reproducibility requirement is incompletely specified. Reproducibility is further complicated by the method of access. Interacting with an LLM through a web interface can yield different results than using an API, as the former may include hidden system prompts or integrated tools like web browsing. In contrast, API access provides a more controlled, researcher-defined environment. These differences can significantly alter a model’s factual accuracy, performance, and reasoning transparency [109,126]. Despite these critical variables, many studies fail to report the specific model version, offering only generic descriptions that limit transparency and hinder replication. Therefore, to ensure transparency and reproducibility in GenAI-based clinical trials, reports should specify the exact model version, the date of use, and the access method (eg, API or web interface) [127].

Two further conditions determine what a GenAI intervention produces, and neither is requested by any CONSORT-AI item. The system prompt establishes the conditions under which a model generates output, and it can shift reading level, enforce consistency across participants, and suppress content the investigators do not intend the intervention to produce [43,46,47]. The inference settings determine how far output may vary under those terms. Their influence is comparable to that of the model version itself. In clinical summarization, adding instruction prompts improves performance but also increases performance variance, so that prompts sharing similar meanings yield markedly different summaries [128]. In a simulation study in which 4 emergency medicine cases were each submitted to GPT-4o 250 times at 5 temperature settings, leading diagnostic accuracy with physical examination data fell from 100% at a temperature of 0.0% to 89.4% at 1.0, while the mean number of distinct diagnoses generated across repetitions rose from 4.5 to 26.25 [129]. Two trials evaluating the same model at different inference settings and system prompts are not evaluating the same intervention at all.

Taken together, these conditions define what would have to be reported for a GenAI intervention to be reproduced: the base model and any auxiliary components, the version and the date on which it was used, the method of access, the system prompt, and the inference parameters under which output was generated. Every one of these is requested by at least one of the 2 reporting frameworks related to GenAI [21,22]. Of these elements, CONSORT-AI presently requests one model version. A trial article reporting only the model version has satisfied Item 5(i) while leaving the intervention unidentifiable. Where an item can be met without its reproducibility being served, the requirement is incompletely specified rather than poorly reported, and revision for GenAI trials merits consideration. Table S17 in Multimedia Appendix 8 consolidates the challenges identified throughout this discussion, distinguishing those arising from items whose underlying construct does not fit a generative intervention from those for which no corresponding item exists.

Almost every element our analysis identifies as missing or unaskable is already established elsewhere: TRIPOD-LLM [21] specifies model name, version, training date, architecture, fine-tuning, and alignment; MI-CLAIM-GEN [22] specifies base and auxiliary components together with structured privacy and security reporting. However, neither framework was developed specifically for randomized trials. These findings identify candidate domains for an extension of CONSORT-AI that would import these settled requirements into the trial setting, restating the input-data items at both the interface and backend levels, replacing the single algorithm version with a full intervention specification, requiring that inadmissible input and the contractual arrangement governing it be defined in advance under national regimes, and naming GenAI-specific error classes under Item 19. So defined, the extension would make reproducible reporting achievable for the class of intervention that now accounts for the great majority of GenAI trials, without duplicating what the wider reporting ecosystem has already settled.

Several limitations of this study should be noted. First, no validated instrument exists for item-level CONSORT-AI assessment, so we developed signaling questions ourselves. Although each question was derived directly from an explicit reporting requirement stated in the CONSORT-AI statement, they have not been externally validated, and a different operationalization could yield different adherence estimates. In particular, requiring all signaling questions to be satisfied for an item to be classified as fully reported is a conservative rule, and items comprising a greater number of requirements are correspondingly less likely to meet it. That said, all assessments were performed independently in duplicate, and item-specific agreement statistics are reported in Table S5 in Multimedia Appendix 4. Second, we restricted inclusion to peer-reviewed, English-language studies explicitly identified as RCTs. This preserved methodological homogeneity but may have excluded randomized evaluations described using other terminology, published in other languages, or available only as preprints, and the excluded studies may differ systematically in reporting practice. Finally, the trials reviewed were predominantly evaluations of text-based language models. As multimodal interventions increasingly enter clinical trials, they will inevitably raise new methodological and reporting challenges that the present CONSORT-AI checklist does not anticipate. To support the continuous updating of these reporting standards, we have prospectively identified and will continue to monitor relevant preprints, trial registrations, and conference abstracts.

### Conclusion

RCTs of GenAI for medical purposes adhere unevenly to CONSORT-AI. Trial metadata and output specification were reported by most trial reports, whereas input data, technical execution, and reproducibility items were reported by few, and handling of poor-quality or missing input data was not reported by any trial. These deficiencies are of 2 kinds. Some reflect nonadherence to requirements that already exist and could be addressed by wider dissemination of the current guideline, while others reflect a mismatch between what the checklist asks and how a generative intervention is specified and delivered and cannot be resolved by adherence alone. Development of a formal reporting extension would require a structured consensus process, as recommended for the development of health research reporting guidelines [130] and as followed in the development of CONSORT-AI itself [12], involving trialists, clinicians, regulators, AI developers, and reporting guideline methodologists. This study establishes that basis, providing the first item-level account of how GenAI trials report against CONSORT-AI and suggesting what such an extension would have to specify to make these trials reproducible and interpretable.

## Supplementary material

10.2196/94543 Multimedia Appendix 1 S1 Comparison of registered protocol.

10.2196/94543 Multimedia Appendix 2 Search strategy.

10.2196/94543 Multimedia Appendix 3 Data extraction form.

10.2196/94543 Multimedia Appendix 4 S5 item-specific agreement statistics.

10.2196/94543 Multimedia Appendix 5 Characteristics of the included reports.

10.2196/94543 Multimedia Appendix 6 Signaling question sensitivity analysis.

10.2196/94543 Multimedia Appendix 7 Subgroup analysis.

10.2196/94543 Multimedia Appendix 8 S17 Characteristics of Gen AI interventions identified in this review.

10.2196/94543 Checklist 1 PRISMA 2020 checklist.

10.2196/94543 Checklist 2 SWiM checklist.

## Abbreviations

BAA: Business Associate Agreement
CENTRAL: Cochrane Central Register of Controlled Trials
CONSORT: Consolidated Standards of Reporting Trials
CONSORT-EHEALTH: Consolidated Standards of Reporting Trials of Electronic and Mobile Health Apps and Online Telehealth
FDR: false discovery rate
FM: foundation model
GDPR: General Data Protection Regulation
GenAI: generative AI
HIPAA: Health Insurance Portability and Accountability Act
LLM: large language model
MI-CLAIM-GEN: Minimum Information about Clinical AI Checklist for Generative Modeling Research
MI-CLEAR-LLM: Minimum Reporting Items for Clear Evaluation of Accuracy Reports of Large Language Models in Health Care
PABAK: prevalence-adjusted bias-adjusted Kappa
PHI: protected health information
PRISMA: Preferred Reporting Items for Systematic Reviews and Meta-Analyses
PRISMA-S: Preferred Reporting Items for Systematic Reviews and Meta-Analyses literature search extension
PROSPERO: International Prospective Register of Systematic Reviews
RAG: retrieval-augmented generation
RCT: randomized controlled trial
SAGER: Sex and Gender Equity in Research
SPIRIT: Standard Protocol Items: Recommendations for Interventional Trials
SQUIRE: Standards for Quality Improvement Reporting Excellence
STROBE: Strengthening the Reporting of Observational Studies in Epidemiology
TRIPOD-LLM: Transparent reporting of a multivariable model for individual prognosis or diagnosis-LLM
