# Supplementary material for: Reporting Gaps and Challenges in Randomized Trials of Generative AI for Medical Use: Insights From a Systematic Review
Source: J Med Internet Res. 2026 Oct 28;28:e94543. doi: 10.2196/94543 (PMC13718382; doi:10.2196/94543)
Supplement: Supplementary file 2 [file jmir-v28-e94543-s010.pdf]

Checklist 2. Synthesis Without Meta-analysis (SWiM) reporting items

SWiM is intended to complement and be used as an extension to PRISMA			
SWiM reporting item	Item description	Page in manuscript where item is reported	Other[a]
Methods			
1 Grouping studies for synthesis	1a) Provide a description of, and rationale for, the groups used in the synthesis (e.g., groupings of populations, interventions, outcomes, study design)	Page 4	
	1b) Detail and provide rationale for any changes made subsequent to the protocol in the groups used in the synthesis	Table S1 in Multimedia Appendix 1	
2 Describe the standardised metric and transformation methods used	Describe the standardised metric for each outcome. Explain why the metric(s) was chosen, and describe any methods used to transform the intervention effects, as reported in the study, to the standardised metric, citing any methodological guidance consulted	Page 4	
3 Describe the synthesis methods	Describe and justify the methods used to synthesise the effects for each outcome when it was not possible to undertake a meta-analysis of effect estimates	Page 4	
4 Criteria used to prioritise results for summary and synthesis	Where applicable, provide the criteria used, with supporting justification, to select the particular studies, or a particular study, for the main synthesis or to draw conclusions from the synthesis (e.g., based on study design, risk of bias assessments, directness in relation to the review question)	Not applicable	No prioritization of studies or results was performed based on risk of bias or study design; all eligible studies were evaluated across applicable checklist items.
5 Investigation of heterogeneity in reported effects	State the method(s) used to examine heterogeneity in reported effects when it was not possible to undertake a meta-analysis of effect estimates and its extensions to investigate heterogeneity	Page 4-5	
6 Certainty of evidence	Describe the methods used to assess certainty of the synthesis findings	Not applicable	Formal assessment of the certainty of evidence was not performed because this review evaluated reporting completeness and checklist adherence rather than synthesizing clinical intervention effect estimates.
7 Data presentation methods	Describe the graphical and tabular methods used to present the effects (e.g., tables, forest plots, harvest plots).<br>Specify key study characteristics (e.g., study design, risk of bias) used to order the studies, in the text and any tables or graphs, clearly referencing the studies included	Page 4-5	
Results			
8 Reporting results	For each comparison and outcome, provide a description of the synthesised findings, and the certainty of the findings. Describe the result in language that is consistent with the question the synthesis addresses, and indicate which studies contribute to the synthesis	Page 10-12	Synthesised adherence findings and subgroup comparisons are presented on Pages 10–12 and Tables S8–S16. Assessment of the

			certainty of findings is not applicable because no intervention effect estimates were synthesized.
Discussion			
9 Limitations of the synthesis	Report the limitations of the synthesis methods used and/or the groupings used in the synthesis, and how these affect the conclusions that can be drawn in relation to the original review question	Page 17	

[a]If the information is not provided in the systematic review, give details of where this information is available (e.g., protocol, other published papers (provide citation details), or website (provide the URL)).
