# Supplementary material for: Reporting Gaps and Challenges in Randomized Trials of Generative AI for Medical Use: Insights From a Systematic Review
Source: J Med Internet Res. 2026 Oct 28;28:e94543. doi: 10.2196/94543 (PMC13718382; doi:10.2196/94543)
Supplement: Supplementary file 3 [file jmir-v28-e94543-s001.docx]

Table S1. Comparison of the registered protocol with the methods implemented

| Domain | As registered (PROSPERO CRD420251034690) | As implemented | Rationale | Timing |
| --- | --- | --- | --- | --- |
| Eligibility criteria (Population) | Studies involving patients or healthcare professionals, in which generative AI was used for medical purposes | Additionally included laypeople, provided the generative AI intervention was evaluated for a medical purpose within a clinical context | A substantial proportion of generative AI trials evaluate layperson-facing tools; restricting the population to patients and healthcare professionals would have excluded a large and clinically relevant part of the evidence base | Before screening |
| Information sources  (Database searching) | Restricted to 2025 | Searched to April 3, 2026 | The number of generative AI trials increased rapidly during the review period, and the registered end date no longer reflected the available evidence | Before screening |
| Information sources (Citation searching) | Not specified | Backward and forward citation searching of included studies was performed on August 12, 2026 (restricted to studies published or available online before April 3, 2026) | To ensure sufficient literature and identify eligible trials that might have been missed by literature database indexing alone | During peer review revision |
| Search strategy | Free-text terms for generative AI | Added MeSH and EMTREE terms for generative AI introduced after registration, expanded terms to named models and related architectures, and replaced the trial filter with the sensitivity-maximizing version of the Cochrane Highly Sensitive Search Strategy | Controlled vocabulary for generative AI became available after registration, and improvement of sensitivity of identifying randomized trails | Before screening |
| Rating categories | A three-level categorization was noted | Four categories: reported, partially reported, not reported, and not applicable | Extraction identified items that could not apply to a given intervention; distinct from items that were applicable but not reported | During data extraction |
| Statistical analysis (subgroup comparisons) | T test of Mann–Whitney U test for two groups, and ANOVA or Kruskal-Wallis test for three or more groups, applied to overall adherence scores | Fisher exact test for two groups and the Freeman–Halton extension for three or more groups, applied to item-level adherence. Benjamini–Hochberg adjusted q values calculated for all subgroup comparisons | The unit of analysis from a study-level composite score to item-level adherence, the tests for continuous adherence outcomes were no longer applicable. Exact tests were used for small cell frequencies. To assess the robustness of item-level comparisons, given the number of tests performed | After data extraction, before examination of subgroup results |
| Inter-rater reliability | Not specified | Prevalence and bias adjusted kappa (PABAK) reported alongside Cohen kappa | Reporting distributions were highly skewed for several items, producing prevalence paradoxes in Cohen kappa | After data extraction, before examination of results |
| Sensitivity analysis | Not specified | Adherence recalculated as the mean proportion of applicable signaling questions satisfied per item | To examine the influence of the all-or-none scoring rule | After examination of results |
