# Supplementary material for: Reporting Gaps and Challenges in Randomized Trials of Generative AI for Medical Use: Insights From a Systematic Review
Source: J Med Internet Res. 2026 Oct 28;28:e94543. doi: 10.2196/94543 (PMC13718382; doi:10.2196/94543)
Supplement: Supplementary file 4 [file jmir-v28-e94543-s002.docx]

Table S2. Search strategy

| Database | Search strategy |
| --- | --- |
| PubMed | (("Generative Artificial Intelligence"[Mesh] OR "Large Language Models"[Mesh] OR "Natural Language Processing"[Mesh] OR "Autoencoder"[Mesh] OR "Generative Adversarial Networks"[Mesh] OR "Generative AI"[tiab] OR "Gen AI"[tiab] OR "GenAI"[tiab] OR "language generation"[tiab] OR "text generation"[tiab] OR "image generation"[tiab] OR "ChatGPT"[tiab] OR "GPT-*"[tiab] OR GPT[tiab] OR GPT3[tiab] OR GPT4[tiab] OR GPT5[tiab] OR GPTs[tiab] OR "LLM"[ti] OR "LLMs"[ti] OR "Large Language Model*"[tiab] OR "Llama"[tiab] OR "Claude"[tiab] OR "Gemini"[tiab] OR "Stable Diffusion"[tiab] OR "AMIE"[tiab] OR "DeepSeek"[tiab] OR "Copilot"[tiab] OR "GAN"[ti] OR "NLP"[ti] OR "Chatbot*"[tiab]) AND ("randomized controlled trial"[pt] OR "controlled clinical trial"[pt] OR "randomized"[tiab] OR "placebo"[tiab] OR "drug therapy"[sh] OR "randomly"[tiab] OR "trial"[tiab] OR "groups"[tiab])) NOT ("animals"[mh] NOT "humans"[mh]) |
| Embase | ('generative artificial intelligence'/exp OR 'large language model'/exp OR 'natural language processing'/exp OR 'autoencoder'/exp OR 'generative adversarial network'/exp OR 'generative ai':ti,ab,kw OR 'gen ai':ti,ab,kw OR 'genai':ti,ab,kw OR 'language generation':ti,ab,kw OR 'text generation':ti,ab,kw OR 'image generation':ti,ab,kw OR 'chatgpt':ti,ab,kw OR 'gpt*':ti,ab,kw OR 'llm':ti OR 'llms':ti OR 'large language model*':ti,ab,kw OR 'llama':ti,ab,kw OR 'claude':ti,ab,kw OR 'gemini':ti,ab,kw OR 'stable diffusion':ti,ab,kw OR 'amie':ti,ab,kw OR 'Copilot':ti,ab,kw OR 'DeepSeek':ti,ab,kw OR 'gan':ti OR 'nlp':ti OR 'chatbot*':ti,ab,kw) AND (('randomized controlled trial'/exp OR 'controlled clinical trial'/de OR random*:ti,ab,tt OR 'randomization'/de OR 'intermethod comparison'/de OR placebo:ti,ab,tt OR compare:ti,tt OR compared:ti,tt OR comparison:ti,tt OR ((evaluated:ab OR evaluate:ab OR evaluating:ab OR assessed:ab OR assess:ab) AND (compare:ab OR compared:ab OR comparing:ab OR comparison:ab)) OR (open NEXT/1 label):ti,ab,tt OR ((double OR single OR doubly OR singly) NEXT/1 (blind OR blinded OR blindly)):ti,ab,tt OR 'double blind procedure'/de OR (parallel NEXT/1 group*):ti,ab,tt OR crossover:ti,ab,tt OR 'cross over':ti,ab,tt OR ((assign* OR match OR matched OR allocation) NEAR/6 (alternate OR group OR groups OR intervention OR interventions OR patient OR patients OR subject OR subjects OR participant OR participants)):ti,ab,tt OR assigned:ti,ab,tt OR allocated:ti,ab,tt OR (controlled NEAR/8 (study OR design OR trial)):ti,ab,tt OR volunteer:ti,ab,tt OR volunteers:ti,ab,tt OR 'human experiment'/de OR trial:ti,tt) NOT ((((random* NEXT/1 sampl* NEAR/8 ('cross section*' OR questionnaire* OR survey OR surveys OR database OR databases)):ti,ab,tt) NOT ('comparative study'/de OR 'controlled study'/de OR 'randomised controlled':ti,ab,tt OR 'randomized controlled':ti,ab,tt OR 'randomly assigned':ti,ab,tt)) OR ('cross-sectional study'/de NOT ('randomized controlled trial'/exp OR 'controlled clinical study'/de OR 'controlled study'/de OR 'randomised controlled':ti,ab,tt OR 'randomized controlled':ti,ab,tt OR 'control group':ti,ab,tt OR 'control groups':ti,ab,tt)) OR ('case control*':ti,ab,tt AND random*:ti,ab,tt NOT ('randomised controlled':ti,ab,tt OR 'randomized controlled':ti,ab,tt)) OR ('systematic review':ti,tt NOT (trial:ti,tt OR study:ti,tt)) OR (nonrandom*:ti,ab,tt NOT random*:ti,ab,tt) OR 'random field*':ti,ab,tt OR ('random cluster' NEAR/4 sampl*):ti,ab,tt OR (review:ab AND review:it) NOT trial:ti,tt OR ('we searched':ab AND (review:ti,tt OR review:it)) OR 'update review':ab OR (databases NEAR/5 searched):ab OR ((rat:ti,tt OR rats:ti,tt OR mouse:ti,tt OR mice:ti,tt OR swine:ti,tt OR porcine:ti,tt OR murine:ti,tt OR sheep:ti,tt OR lambs:ti,tt OR pigs:ti,tt OR piglets:ti,tt OR rabbit:ti,tt OR rabbits:ti,tt OR cat:ti,tt OR cats:ti,tt OR dog:ti,tt OR dogs:ti,tt OR cattle:ti,tt OR bovine:ti,tt OR monkey:ti,tt OR monkeys:ti,tt OR trout:ti,tt OR marmoset*:ti,tt) AND 'animal experiment'/de) OR ('animal experiment'/de NOT ('human experiment'/de OR 'human'/de)))) AND 'human'/de NOT ('conference abstract'/it OR 'conference paper'/it OR 'editorial'/it OR 'letter'/it) |
| CENTRAL | (([mh "Generative Artificial Intelligence"] OR [mh "Large Language Models"] OR [mh "Natural Language Processing"] OR [mh "Autoencoder"] OR [mh "Generative Adversarial Networks"] OR "generative NEXT AI":ti,ab,kw OR "gen NEXT AI":ti,ab,kw OR genai:ti,ab,kw OR "language NEXT generation":ti,ab,kw OR "text NEXT generation":ti,ab,kw OR "image NEXT generation":ti,ab,kw OR chatgpt:ti,ab,kw OR gpt*:ti,ab,kw OR llm:ti OR llms:ti OR "large NEXT language NEXT model*":ti,ab,kw OR llama:ti,ab,kw OR claude:ti,ab,kw OR gemini:ti,ab,kw OR "stable NEXT diffusion":ti,ab,kw OR amie:ti,ab,kw OR deepseek:ti,ab,kw OR copilot:ti,ab,kw OR gan:ti OR nlp:ti OR chatbot*:ti,ab,kw) AND ([mh "randomized controlled trial"] OR [mh "controlled clinical trial"] OR randomized:ti,ab,kw OR placebo:ti,ab,kw OR [mh "drug therapy"] OR randomly:ti,ab,kw OR trial:ti,ab,kw OR groups:ti,ab,kw)) NOT ([mh animals] NOT [mh humans]) |
