# Supplementary material for: Reporting Gaps and Challenges in Randomized Trials of Generative AI for Medical Use: Insights From a Systematic Review
Source: J Med Internet Res. 2026 Oct 28;28:e94543. doi: 10.2196/94543 (PMC13718382; doi:10.2196/94543)
Supplement: Supplementary file 5 [file jmir-v28-e94543-s003.docx]

Table S3. Definitions of classification codes for data extraction

| Item | Description | | | |
| --- | --- | --- | --- | --- |
| Classification code | Code | Rating | Definition at signaling question^a^ level | Examples |
|  | 1 | Reported | The specific reporting requirement specified in the CONSORT-AI statement and explanation is fully reported. | Item 5(i) signaling question 1: The exact model release snapshot and access method are specified (e.g., "GPT-4-0613 accessed via Azure OpenAI API on May 12, 2024"). |
|  | 2 | Not reported | The specific signaling question is not addressed at all in the text, tables, protocols, or registration details. | Item 5(iii) signaling question 1: Failure to report quantitative amount of low-quality, uninterpretable, or missing input prompts. |
|  | 3 | Partially reported | The concept or reporting area in question is mentioned, but essential methodological or operational elements required for full reproducibility are missing. | Item 5(i) signaling question 1: Mentions only a general model name (e.g., "ChatGPT" or "GPT-4") without reporting the snapshot version, access date, or interface type (web vs. API). |
|  | 4 | Not applicable | The signaling question cannot be logically applied to the clinical trial design or intervention structure, or the conditional prerequisites are not met. | Item 4a(ii) signaling question 2-4: For clinical trials evaluating real-time conversational interactions where external datasets were not pre-constructed or curated prior to randomization. |
| Reference | Sentences or paragraphs that form the basis for the evaluation (page and section titles if too long) | | | |
| Note | Instructions of references | | | |
| Additional comments | Any additional comments (e.g., if reported in the included studies and considered important to report) | | | |

^a^Handling rules for conditional signaling questions: When a primary prerequisite condition did not occur in the trial (e.g., the algorithm version remained constant throughout the trial for Item 5(i); no site-specific modifications were required for Item 4b), all dependent follow-up signaling questions were assigned "Not applicable (Code 4)" and excluded from the item-level evaluation. When performance error analysis was conducted, signaling questions evaluating methods, error cases, implications of error, and mitigation strategies were rated, while the justification question for omission was coded as “Not applicable”. When error analysis was not conducted, signaling questions evaluating its execution were coded as Not applicable only upon the provision of a valid justification for omitting the analysis.

Table S4. Data extraction form

| Section/Topic | No | CONSORT-AI checklist | No | Signaling questions | Classification code | Final  classification code | Reference | Note | Additional comments |
| --- | --- | --- | --- | --- | --- | --- | --- | --- | --- |
| Title and Abstract |  |  |  |  |  |  |  |  |  |
| Title and structured abstract | 1a, b (ⅰ) | Indicate that the intervention involves artificial intelligence/machine learning in the title and/or abstract and specify the type of model. | 1 | Is it stated in the title and/or abstract that the intervention involves AI/ML? |  |  |  |  |  |
|  |  |  | 2 | Are the specific terminology relating model type and architecture detailed in the abstract? |  |  |  |  |  |
|  | 1a, b (ⅱ) | State the intended use of the AI intervention within the trial in the title and/or abstract. | 1 | Is the intended use of the AI intervention stated in the title and/or abstract? |  |  |  |  |  |
|  |  |  | 2 | Is the disease context described in the title and/or abstract? |  |  |  |  |  |
| Open science |  |  |  |  |  |  |  |  |  |
| Data sharing | 25 | State whether and how the AI intervention and/or its code can be accessed, including any restrictions to access or re-use. | 1 | Is it clearly reported whether and how the AI intervention and/or its code can be accessed or re-used? |  |  |  |  |  |
| Introduction |  |  |  |  |  |  |  |  |  |
| Background and rationale | 2a (ⅰ) | Explain the intended use of the AI intervention in the context of the clinical pathway, including its purpose and its intended users (for example, healthcare professionals, patients, public). | 1 | Is the intended use of the AI intervention within the clinical pathway included? |  |  |  |  |  |
|  |  |  | 2 | Is the intended user of the AI intervention included? |  |  |  |  |  |
| Methods |  |  |  |  |  |  |  |  |  |
| Trial setting | 4b | Describe how the AI intervention was integrated into the trial setting, including any onsite or offsite requirements. | 1 | Are the hardware and software requirements described? (e.g. vendor-specific device, computing hardware, cloud integration at each site) |  |  |  |  |  |
|  |  |  | 2 | If site-specific changes were required, is the process clearly described (e.g. fine-tuning on local data)? |  |  |  |  |  |
| Eligibility criteria | 4a (ⅰ) | State the inclusion and exclusion criteria at the level of participants. | 1 | Are the inclusion and exclusion criteria at the participant level stated? |  |  |  |  |  |
|  | 4a (ⅱ) | State the inclusion and exclusion criteria at the level of the input data. | 1 | Are the minimum requirements (e.g. image resolution, quality metrics or data format) for the input data specified at the input level? |  |  |  |  |  |
|  |  |  | 2 | Is it stated when the input data eligibility was assessed? |  |  |  |  |  |
|  |  |  | 3 | Is it stated how the input data eligibility was assessed? |  |  |  |  |  |
|  |  |  | 4 | Is it stated by whom the input data eligibility was assessed? |  |  |  |  |  |
| Intervention and comparator | 5 (ⅰ) | State which version of the AI algorithm was used. | 1 | Is the version of the AI system used stated? |  |  |  |  |  |
|  |  |  | 2 | Is it stated whether the same version was used as in previous supporting studies? |  |  |  |  |  |
|  |  |  | 3 | Is it stated whether the version changed during the trial? |  |  |  |  |  |
|  |  |  | 3-1 | If the version changed, are the changes between versions described? |  |  |  |  |  |
|  |  |  | 3-2 | If the version changed, are the rationales for the changes described? |  |  |  |  |  |
|  |  |  | 4 | If available, is a regulatory marking reference (e.g. unique device identifier) provided? |  |  |  |  |  |
|  | 5 (ⅱ) | Describe how the input data were acquired and selected for the AI intervention. | 1 | Is it described how the input data were acquired for the AI intervention? |  |  |  |  |  |
|  |  |  | 2 | Is it described how the input data were selected for the AI intervention? |  |  |  |  |  |
|  |  |  | 3 | Is it described how the input data were pre-processed before analysis by the AI system? |  |  |  |  |  |
|  | 5 (ⅲ) | Describe how poor quality or unavailable input data were assessed and handled. | 1 | Is the amount of poor quality or missing data reported? |  |  |  |  |  |
|  |  |  | 2 | Is it described how the poor quality or missing data were identified? |  |  |  |  |  |
|  |  |  | 3 | Is it described how the poor quality or missing data were handled? |  |  |  |  |  |
|  |  |  | 4 | Is any minimum quality standard for the input data stated? |  |  |  |  |  |
|  |  |  | 5 | If the minimum quality was not met, is it stated where the standard was not achieved? |  |  |  |  |  |
|  |  |  | 6 | If the minimum quality was not met, is the handling process described including any impact on the participant care pathway? |  |  |  |  |  |
|  |  |  | 7 | If relevant, is the same information about poor quality or unavailable data reported for the control intervention? |  |  |  |  |  |
|  |  |  | 8 | If applicable, is it stated whether this minimum quality standard differed from the pre-randomization input-data eligibility criteria? |  |  |  |  |  |
|  | 5 (ⅳ) | Specify whether there was human–AI interaction in the handling of the input data, and what level of expertise was required of users. | 1 | Is the human-AI interaction stated? |  |  |  |  |  |
|  |  |  | 2 | Is the human-AI interface when input data handled described? |  |  |  |  |  |
|  |  |  | 3 | Is the required user expertise or any user training/instructions for input data handling described? |  |  |  |  |  |
|  | 5 (ⅴ) | Specify the output of the AI intervention | 1 | Is the output of the AI intervention clearly specified? |  |  |  |  |  |
|  | 5 (ⅵ) | Explain how the AI intervention’s outputs contributed to decision-making or other elements of clinical practice. | 1 | Are downstream interventions that could affect outcomes described? |  |  |  |  |  |
|  |  |  | 2 | Are the required user expertise and any training or instructions for interpreting the outputs described? |  |  |  |  |  |
|  |  |  | 3 | If relevant, is comparable information provided for the comparator intervention? |  |  |  |  |  |
|  |  |  | 4 | Is any discrepancy between intended (as specified in the trial protocol) and actual decision-making reported? |  |  |  |  |  |
| Results |  |  |  |  |  |  |  |  |  |
| Harms | 19 | Describe results of any analysis of performance errors and how errors were identified, where applicable. If no such analysis was planned or done, justify why not. | 1-1 | Is it described how performance errors were identified? |  |  |  |  |  |
|  |  |  | 1-2 | Are the cases of error reported? |  |  |  |  |  |
|  |  |  | 1-3 | Are the implications of any performance-error analysis reported? |  |  |  |  |  |
|  |  |  | 1-4 | Are the risk-mitigation strategies defined? |  |  |  |  |  |
|  |  |  | 2 | If no such analysis was planned or done, was the reason explained? |  |  |  |  |  |
