# Supplementary material for: Reporting Gaps and Challenges in Randomized Trials of Generative AI for Medical Use: Insights From a Systematic Review
Source: J Med Internet Res. 2026 Oct 28;28:e94543. doi: 10.2196/94543 (PMC13718382; doi:10.2196/94543)
Supplement: Supplementary file 6 [file jmir-v28-e94543-s004.docx]

Table S5. Item-specific percent agreements and Kappa statistics

| Item | Summary of CONSORT-AI checklist | $P_{o}$ (%) | $P_{e}$ (%) | Cohen’s κ  (95% CI) | PABAK |
| --- | --- | --- | --- | --- | --- |
| 1a, b(ⅰ) | Indicate that the intervention involves artificial intelligence/machine learning in the title and/or abstract and specify the type of model. | 100.00 | 76.04 | 1.00  (1.00 – 1.00) | 1.00 |
| 1a, b(ⅱ) | State the intended use of the AI intervention within the trial in the title and/or abstract. | 98.65 | 98.65 | 0.00  (0.00 – 0.00) | 0.97 |
| 2a(ⅰ) | Explain the intended use of the AI intervention in the context of the clinical pathway, including its purpose and its intended users (for example, healthcare professionals, patients, public). | 97.30 | 69.28 | 0.91  (0.76 – 1.00) | 0.95 |
| 4a(ⅰ) | State the inclusion and exclusion criteria at the level of participants. | 93.24 | 84.73 | 0.56  (0.14 – 0.85) | 0.90 |
| 4a(ⅱ) | State the inclusion and exclusion criteria at the level of the input data. | 89.19 | 63.81 | 0.70  (0.48 – 0.88) | 0.86 |
| 4b | Describe how the AI intervention was integrated into the trial setting, including any onsite or offsite requirements. | 85.14 | 38.59 | 0.76  (0.61 – 0.88) | 0.78 |
| 5(ⅰ) | State which version of the AI algorithm was used. | 78.38 | 37.71 | 0.65  (0.50 – 0.80) | 0.71 |
| 5(ⅱ) | Describe how the input data were acquired and selected for the AI intervention. | 82.43 | 76.81 | 0.24  (-0.06 – 0.52) | 0.74 |
| 5(ⅲ) | Describe how poor quality or unavailable input data were assessed and handled. | 81.08 | 79.93 | 0.06  (-0.10 – 0.31) | 0.72 |
| 5(ⅳ) | Specify whether there was human–AI interaction in the handling of the input data, and what level of expertise was required of users. | 77.03 | 43.17 | 0.60  (0.42 – 0.75) | 0.69 |
| 5(ⅴ) | Specify the output of the AI intervention | 87.84 | 82.51 | 0.30  (-0.03 – 0.61) | 0.82 |
| 5(ⅵ) | Explain how the AI intervention’s outputs contributed to decision-making or other elements of clinical practice. | 83.78 | 32.62 | 0.76  (0.63 – 0.88) | 0.78 |
| 19 | Describe results of any analysis of performance errors and how errors were identified, where applicable. If no such analysis was planned or done, justify why not. | 77.03 | 32.74 | 0.66  (0.51 – 0.79) | 0.66 |
| 25 | State whether and how the AI intervention and/or its code can be accessed, including any restrictions to access or re-use. | 98.65 | 51.53 | 0.97  (0.91 – 1.00) | 0.97 |
| Overall | All 14 items pooled (1,036 item-level judgments) | 87.84 | 36.30 | 0.81  (0.78 – 0.84) | 0.84 |

Note: $\boldsymbol{P}_{\boldsymbol{o}}$, observed proportion of agreement; $\boldsymbol{P}_{\boldsymbol{e}}$, expected proportion of agreement; CI, confidence interval; PABAK, prevalence- and bias-adjusted Kappa. Low Cohen’s $\boldsymbol{\kappa}$ values relative to high $\boldsymbol{P}_{\boldsymbol{o}}$ (e.g., Item 1a, b (ii) and Item 5 (iii)) reflect the *prevalence paradox*, where highly skewed marginal distributions artificially suppress the $\boldsymbol{\kappa}$ statistic. PABAK values provide the prevalence-adjusted reliability estimates.
