# Supplementary material for: Reporting Gaps and Challenges in Randomized Trials of Generative AI for Medical Use: Insights From a Systematic Review
Source: J Med Internet Res. 2026 Oct 28;28:e94543. doi: 10.2196/94543 (PMC13718382; doi:10.2196/94543)
Supplement: Supplementary file 7 [file jmir-v28-e94543-s005.docx]

Table S6. Study characteristics of included reports

| Author | Year | Country | Total sample | Study design | Intended use | Intended user  (Interacting user) | Type of Gen AI | Outcome measurements |
| --- | --- | --- | --- | --- | --- | --- | --- | --- |
| Cheng, S. T. & Ng, P. H. F. [1] | 2026.01 | China | 174 caregivers | 2 parallel arms  (PDC30, Waitlist) | To deliver psychoeducation for dementia caregivers | Dementia caregivers | Positive Dementia Caregiving in 30 Days (PDC30, powered by GPT-4o) | PHQ-9  Abbreviated Profile of Mood States, Anxiety Subscale (APOMS-A)  Zarit Burden Interview, brief version (ZBI-B)  Positive Aspects of Caregiving Scale-II (PACS-II) |
| Cheng, S. T. & Ng, P. H. F. [2] | 2026.02 | China | 174 caregivers | 2 parallel arms  (PDC30, Waitlist) | To deliver psychoeducation for dementia caregivers | Dementia caregivers | PDC30 (powered by GPT-4o) | PHQ-9  Zarit Burden Interview, brief version (ZBI-B)  Positive Aspects of Caregiving Scale-II (PACS-II)  Revised Scale for Caregiving Self-Efficacy  Reappraisal coping (Brief COPE) |
| Zhao, H. [3] | 2026.01 | China | 1,068 patients | 2 parallel arms  (GenDSA-V2, Standard clinical protocols) | To reduce intra-operative radiation exposure during interventional procedures by acquiring digital subtraction angiography (DSA) images at a lowered frame rate and reconstructing them into full-rate, diagnostic-quality videos in real time | No interaction required | GenDSA-V2 | Radiation dose  Operation time  Intra-operative complications  Subjective scores of video quality and diagnostic capabilities  Overall quality and diagnostic capacity of 2D-DSA images  Accuracy of generated DSA sequences |
| Cao, Y. et al. [4] | 2024.12 | China | 29 patients | 2 parallel arms  (Baichuan 2 group, GPT-4.0 group) | Glaucoma patient education | Glaucoma patients | Baichuan 2, ChatGPT 4.0 | Patient: satisfaction, readability, and helpfulness  Six certiﬁcated ophthalmologists: correctness, completeness, readability, helpfulness, and safety |
| Goh, E. et al. [5] | 2024.10 | US | 50 physicians | 3 parallel arms  (Physicians with GPT, Physicians with conventional resources, ChatGPT only) | Physicians’ diagnostic reasoning | Physicians | ChatGPT Plus (GPT-4) | Diagnostic performance  Time spent per case |
| Wan, P. et al. [6] | 2024.10 | China | 2,164 patients | 2 parallel arms  (Nurse-SSPEC group, Nurse only group) | LLM to assist communication in medical center reception sites | Outpatients | SSPEC (powered by fine-tuned GPT-3.5) | Patient satisfaction with the response  Incidence of repeated Q&Rs and negative emotions  Response quality in terms of factuality, integrity, safety, empathy and readability  Nurse satisfaction feedback in collaborating with the SSPEC  Performance of the alert system |
| Baker, Hayden P. et al. [7] | 2024.02 | US | 11  (4 medical students, and physicians) | 3 crossover arms  (Typing, Dictation, ChatGPT) | LLM to document history of present illness (HPI) of standardized patient histories | Physicians and medical students | ChatGPT | Time to complete the HPI  Word count  Documentation quality (PDQI-9)  Number of history items included in the documentation  Overall global assessment of rating (5-likert)  Nonfactual items (hallucination, incorrect history points) |
| McDuff, D. et al. [8] | 2025.04 | US | 20 physicians | 2 parallel arms  (Physician with assistance from AMIE, Physicians with assistance from search engines and standard medical resources) | Generating a differential diagnosis (DDx) and diagnostic reasoning | Physicians | Articulate Medical Intelligence Explorer (AMIE, powered by PaLM 2) | Top-1 and top-10 accuracy of DDx lists  Comprehensiveness  Appropriateness  Quality score from generalist physicians by specialists  Task duration  Length of DDx lists |
| Gorenshtein, A. et al. [9] | 2025.08 | Israel | 200 patients | 3 parallel arms  (AI+physician, Physician soley, Fully automated AI-report) | Generating electrodiagnostic report | Physician | AI framework (INSPIRE, powered by GPT-4o) | Electrodiagnostic report quality  Findings and Clinical Diagnosis scores  quality of physician–AI collaboration  final satisfaction questionnaire |
| Wu, Y. et al. [10] | 2025.11 | China | 75 patients | 2 parallel arms  (ChatMyopia, Traditional leaflet) | For myopia-related consultation in primary eye care settings | Patients seeking information related to myopia care | ChatMyopia (powered by Mistral-123B) | Chinese version of the Medical Interview Satisfaction Scale-Revised (C-MISS-R)  Patients’ perceptions  10-item Decision Conflict Scale |
| Ke, Y. H. et al. [11] | 2025.07 | Singapore | 14 physicians | 2 crossover arms  (Utilizing PEACH, Standard workflows without AI assistance) | Clinical decision support system in preoperative consultations | Physician | PEACH (powered by Claude 3.5 Sonnet) | Clinical efficiency  Documentation quality  Safety and accuracy  User acceptance  Economic impact |
| Tailor, P. D. et al. [12] | 2025.04 | US | 1,068 patients | 2 parallel arms  (Standard ophthalmology note with LLM-generated plain language summaries, Standard ophthalmology note only) | Generating plain language summaries integrated into standard ophthalmology notes in improving diagnostic understanding, satisfaction, and clarity | Not reported | Mixtral 8x7B; Mistral AI | Non-ophthalmology provider survey questions  Readability  Semantic similarity  Sentence transformers Lexical overlap metrics |
| Ye, X. et al. [13] | 2025.09 | China | 40 secondary school students | 2 parallel arms  (WarmGPT, Information control condition) | In alleviating negative emotions and enhancing overall mental health in secondary school students | Secondary school student | WarmGPT (powered by GPT-4-0314)  (temperature 0.5, maximum length 512, top-p 0.85, frequency penalty 0.3, presence penalty 0.3) | Children's Depression Inventory (CDI)  Screen for Child Anxiety-Related Emotional Disorders (SCARED)  Positive and Negative Scale for Children (PANAS-C) |
| Sabour, S. et al. [14] | 2023.05 | China | 247 general population | 3 parallel arms  (CBT-based Emohaa, Full Emohaa, Waitlist) | For improving symptoms of mental distress, specifically depression, anxiety, negative affect, and insomnia | General population | Emohaa | Patient Health Questionnaire (PHQ-9)  Generalized Anxiety Disorder (GAD-7)  20-item Positive and Negative Affect Schedule (PANAS)  7-item Insomnia Severity Index (ISI) |
| Aydin, F. et al. [15] | 2023.11 | Türkiye | 139 patients | 2 parallel arms  (AI Chatbot, Conventional method) | For obtaining informed consent from patients who will undergo coronary angiography | Patients who were given a coronary angiography decision | ChatGPT 3 | Satisfaction questionnaire (5-likert)  Patients’ awareness of coronary angiography procedure and risk questionnaire |
| Tai-Seale, M. et al. [16] | 2024.04 | US | 122 physicians | 3 parallel arms  (Immediate activation, Delayed activation, No activation) | GenAI-drafted replies for patient messages | Physicians | GPT-4 | Time spent reading messages  Time spent replying to messages  Length of replies  Physician likelihood to recommend GenAI drafts |
| Yahagi, M. et al. [17] | 2024.10 | Japan | 85 patients | 2 parallel arms  (ChatGPT, standard information from anesthesia nurses) | Mitigating preoperative anxiety related to general anesthesia by addressing patient inquiries | Patient scheduled for elective surgery under general anesthesia | ChatGPT-3.5 | State-Trait Anxiety Inventory (STAI) self-report questionnaire  participant satisfaction with the provided information  improvement in patients’ understanding of the treatment process  the perception of ChatGPT’s responses as more relevant than those of the nurses |
| Chung, D. et al. [18] | 2024.12 | Canada | 20 patients | 2 parallel arms  (ChatGPT exposure preconsultation, Direct physician consultation) | Pre-vasectomy counselling | Patients who were interested in undergoing a vasectomy | ChatGPT-4 | Length of visit  Number of questions  Understanding of procedure, risks, benefits  Predication for patients having used ChatGPT  Patient-reported outcomes of quality of clinical encounter  Qualitative survey of patient experiences with ChatGPT for pre-vasectomy counselling  Accuracy of ChatGPT responses to patient questions via retrospective validation |
| Ebnali Harari, R. et al. [19] | 2024.11 | US | 54 general population | 3 parallel arms  (ChatGPT, Supervised-ChatGPT, Paper guide) | Providing clinical guidance during cardiac arrest scenarios | Undergraduate and graduate students with no medical background or prior cardiopulmonary resuscitation experience | ChatGPT 3.5 | Scenario completion time  Decision accuracy  Heart rate (bpm)  LF/HF ratio  root mean square of successive differences (RMSSD)  Perceived cognitive load (NASA/TLX)  The System Usability Scale  Trust in AI |
| Hou, Z. et al. [20] | 2025.04 | China | 2,671 general population | 2 parallel arms  (Chatbot, Usual care) | To improve HPV vaccination uptake among middle school girls | Parents of female students currently enrolled in participating middle schools | HPVchatbot (powered by GPT-4) | The receipt or scheduled appointment of the HPV vaccine  Whether participants had consulted health professionals about getting their daughters vaccinated against HPV in the two-week post-intervention period  Parental willingness to vaccinate their daughters against HPV  HPV vaccination literacy  HPV vaccine confidence  chatbot usage outcomes |
| Lukac, PJ. et al. [21] | 2025.11 | US | 238 physicians | 3 parallel arms  (DAX, Nabla, Usual care) | To record patient encounters and generate visit notes | Physician | Microsoft DAX Copilot 2.0 and Nabla 1.5 | documentation time  Mini-Z 2.0  four-item physician task load  Professional Fulfillment Index  usability, occurrence of inaccuracies and biases, and perceived risks to safety |
| Wu, Y. et al. [22] | 2025.08 | China | 668 patients | 2 parallel arms  (Ophthalmologists with EyeFM, Standard of care screenings) | To serve as a multimodal vision-language clinical copilot that assists eyecare providers in diagnosing and managing common eye diseases | Physician | EyeFM (powered by LLaVa, with an embedded LLaMa 2) | Correct Diagnosis Rate  Correct Referral Rate  Standardization Score of Clinical Reports  Patient Compliance with Self-management  Patient Compliance with Referral Suggestions  Participant Satisfaction |
| Berzolla, E. et al. [23] | 2025.04 | US | 64 patients | 2 parallel arms  (AI translation, Standard MRI) | Translation of an MRI report | Not reported | ChatGPT 4.0 | Comprehension quiz score  Quiz time  Report preference  Clarity rating  Trust rating  Completeness score  Accuracy score  Frequency of AI use  Prior use of AI to interpret or evaluate health information  Likelihood to use AI to interpret health information / MRI reports in the future |
| Kerimoglu Yildiz, G., Turk Delibalta, R. & Coktay, Z. [24] | 2025.05 | Türkiye | 60 mothers | 2 parallel arms  (Breastfeeding counseling mobile application, Breastfeeding counseling booklet) | Breastfeeding counseling | Mothers seeking breastfeeding training at the postpartum services | Not reported (powered by ChatGPT) | Breastfeeding Charting System and Assessment Tool (LATCH)  Postnatal breastfeeding self-efficacy scale  Beck anxiety inventory (BAI) |
| Aydemir, U. [25] | 2025.06 | Türkiye | 26 parents | 2 parallel arms  (ChatGPT, Unspecified control) | For promoting physical activity in children with autism spectrum disorder | Parents of children with autism spectrum disorder | ChatGPT 4.0 | Feasibility Questionnaire  Leisure Time Exercise Questionnaire |
| Gan, W. et al. [26] | 2025.03 | China | 55 patients | 2 parallel arms  (Informed consent assisted by ChatGPT, Traditional informed consent) | Alleviating anxiety in total knee arthroplasty consent process | Physicians | ChatGPT 4.0 (temperature parameter 0.7, top-p parameter 0.9) | HADS, PAS-7, VAS-A, WOMAC, and VAS-P  Two Single-Item Overall Patient Satisfaction Questionnaires (SIOPSQs)  The most frequent queries, accuracy, objectivity, acceptability |
| Chen, C. et al. [27] | 2025.03 | China | 124 general population | 2 parallel arms  (AI chatbot, Nurse hotline) | Reducing anxiety and depression levels in the general population | General population | Not reported | Patient Health Questionnaire (PHQ-9)  Generalized Anxiety Disorder (GAD-7)  Service satisfaction |
| Weuthen, F. A. et al. [28] | 2025.05 | German | 56 medical students and physicians | 2 parallel arms  (ChatGPT, Own research) | To support medical research and clinical decision-making | Medical students and physicians | GPT-4-0125-preview | Case performance  Changes in specific question accuracy  Self-assessed occupational medicine expertise |
| Zhang, H. et al. [29] | 2025.06 | China | 75 patients | 3 parallel arms  (ChatGPT, Ernie bot, Traditional physician education) | Perioperative management of urolithiasis surgery | Patients undergoing urological stone surgery | GPT-4o and Ernie bot 3.5 | State–Trait Anxiety Inventory (STAI)  Number of participant questions asked  Education quality metrics  Postoperative pain measured by Visual Analog Scale (VAS) |
| Akdogan, O. et al. [30] | 2025.03 | Türkiye | 150 patients | 2 parallel arms  (GPT-assisted counseling group, Standard clinician-led education group) | Alleviating anxiety and depression in newly diagnosed cancer patients beginning anti-cancer treatment | Newly diagnosed cancer patients | ChatGPT-4.0 | Changes in anxiety and depression (HADS-A, HADS-D)  Accuracy of AI-generated response  Most frequently asked questions (frequency, percentage) |
| Zhao, Y. et al. [31] | 2025.09 | China | 657 subclinical young adults | 2 parallel arms  (Douyin companion bot, Waitlist) | To intervene in young people's negative emotions | Subclinical young adult | Douyin Xinqing (Douyin companion bot, powered by a fine-tuned Volcano Ark LLM) | Patient Health Questionnaire (PHQ-9)  Generalized Anxiety Disorder (GAD-7)  Positive and Negative Affect Schedule (PANAS) |
| Lai, H. L. et al. [32] | 2025.10 | Taiwan | 80 nurses | 2 parallel arms  (ISCC Chatbot, Standard intervention) | Improving Sleep Quality and Work-Related Outcomes Among Nurses | Nurse | Internet-Support-Coping-Caring with Chatbot (ISCC Chatbot, powered by generative pretrained transformer technology) | Pittsburgh Sleep Quality Index (PSQI)  Perceived stress scale (PSS)  Hospital Anxiety and Depression Scale (HADS) |
| Anand, E. et al. [33] | 2025.12 | UK | 61 patients | 2 parallel arms  (Simple MRI report & AI-generated patient summary, Complex MRI report & AI-generated patient summary) | Generating lay summaries of MRI reports for patients with complex perianal fistulae | Not reported | ChatGPT-4o | Readability  Comprehensibility  Perceived utility  Likelihood of follow-up questions  Trustworthiness  Qualitative feedback |
| Islam, S. [34] | 2025.05 | Pakistan | 100 patients | 2 parallel arms  (AI-generated content, Traditional education) | Generating educational content on patient understanding and anxiety in endodontics and restorative dentistry | Not reported | ChatGPT | Perception of clarity, usefulness, comprehensive, trust, and anxiety  Professional evaluations of the accuracy, thoroughness, and worth of the instructional material. |
| Busch, F. et al. [35] | 2025.03 | Germany | 86 patients | 2 parallel arms  (Patient information assistant, Docter-led informed consent) | For Pre-CT Informed Consent counseling | Patients scheduled for CT imaging | Patient Information Assistant (PIA, powered by ChatGPT4o) | Perceived clarity of information  Reduction of concerns  Level of information  Satisfaction with information  Consultation duration  Preference for consultation  Overall quality  Scientific and clinical evidence  Clinical usefulness and relevance  Consistency  Up-to-dateness of responses |
| Donner, S. et al. [36] | 2025.10 | German | 36 patients | 3 parallel arms  (ChatGPT+ retrieval-augmented generation, Native ChatGPT, Human only) | To assist informed consent in total hip arthroplasty | Patients scheduled for elective, primary total hip arthroplasty | ChatGPT 4o (May 13, 2024) | Patients’ Preferences and Satisfaction Levels (PPSQ)  Health Literacy Test for Total Hip Arthroplasty  General Attitudes Towards Artificial Intelligence Scale (GAAIS)  State-Trait Anxiety Inventory (STAI)  Relevance, accuracy, clarity, completeness, and evidence-based  The level of hallucinations |
| Goh, E. et al. [37] | 2025.02 | US | 92 physicians | 2 parallel arms  (Physicians+GPT-4, Physicians+conventional resources only) | For improvement of physician performance on patient care tasks | Physician | ChatGPT-4 | Total score on expert-developed scoring rubrics  Domain-specific scores  Time spent on cases  All responses for potential harm |
| Stephan, D. et al. [38] | 2025.06 | German | 300 patients | 3 parallel arms  (Original AI generated report, Patient-friendly simplified report, Accessibility optimized report) | Generating radiology reports simplified to improve patient understanding, readability, and communication quality | Not reported | GPT-4 (temperature 0.7, maximum token limit 1500, frequency penalty 0.0, and presence penalty 0.6) | Word count  Sentence count  Proportion of long words  Flesch Reading Ease (FRE) score  LIX readability index |
| Allen, J. W. et al. [39] | 2025.10 | UK | 375 general population | 3 parallel arms  (Consent-GPT, Treating surgeon, Resident doctor) | To aid in surgical consent process | General population | Consent-GPT (powered by GPT-4) | Perceived consent validity  Justified to sue the hospital  Satisfaction with the consent process  Trust that the consent agent will provide all relevant information  Doubt about the treating surgeon's decision to delegate consent  Comfort in sharing potentially embarrassing personal health information  Trust in medically specialized AI for accurate medical advice  Trust in doctors for accurate medical advice |
| Samardzic, D. et al. [40] | 2025.11 | Croatia | 129 patients | 2 crossover arms  (GPT-generated text, Clinician-written text) | Generating discharge texts in emergency ophthalmology | Researcher | GPT-5 (temperature 1.0, top-p 1.0) | Perceived empathy  Clarity of explanation  Usefulness  Trust  Adherence intention  Overall satisfaction  Overall preference |
| Goh, E. et al. [41] | 2025.03 | US | 50 physicians | 2 parallel arms  (White male patient vignette with GPT assisted clinical decision making, Black female patient vignette with GPT assisted clinical decision making) | Artiﬁcial intelligence assistance in clinical decision making | Physicians | ChatGPT Plus (GPT-4) | Accuracy of answers to the clinical decision questions  Variance in accuracy before and after intervention |
| Gupta, A. et al. [42] | 2025.01 | India | 100 patients | 2 parallel arms  (Simplified report, Original report) | Generating simplified radiology reports on oncology patients | Not reported | Claude Opus | Knowledge level about the primary cancer site and extent of their disease  Confidence score about the knowledge  Understandability score  Time taken by the patients to read and interpret the given reports  Consequent psychological distress after reading reports  Number of additional queries raised by the patients  Number of corrections required by the supervising radiologist |
| Indrayanti, I. et al. [43] | 2025.12 | Indonesia | 32 university students | 2 parallel arms  (PsyBot, Neutral activity) | Delivering psychological first aid and reducing student loneliness | University students indicating moderate to high loneliness | PsyBot (powered by GPT-3) | UCLA Loneliness Scale  Loneliness Vulnerability Scale (LVS)  Warwick-Edinburgh Mental Well-Being Scale (WEMWBS)  Coping Scale |
| Campellone, T. R. et al. [44] | 2025.05 | US | 160 general population | 2 parallel arms  (Woebot for mood and anxiety with generative elements, Woebot for mood and anxiety) | To deliver a guided self-help program using cognitive behavioral therapy, psychoeducation, and self-management | General population | Woebot for mood and anxiety with generative elements (powered by text-davinci-003, text-curie-001, and fine-tuned text-embedding-ada-002) | Client Satisfaction Questionnaire-8 (CSQ-8)  Working Alliance Inventory-Short Revised Bond subscale (WAI-SR Bond)  Empathic listening and reflection success  User engagement  Adverse events  Instances of concerning language detected in user free-text inputs  Posttrial technical guardrail assessment success rate  Participant exposure to and sentiment toward AI |
| Tu, T. et al. [45] | 2025.04 | US | 20 validated patient-actors | 2 crossover arms  (DDx by AMIE, DDx by primary care physicians) | Conversational DDx (clinical history-taking and diagnostic dialogue) | Patient-actors | AMIE (powered by PaLM 2) | DDx accuracy (Specialist-rated top-k diagnostic accuracy)  Conversation qualities by patient actor (GMCPQ, PACES, PCCBP)  Conversation and reasoning qualities by specialist physician (PACES, PCCBP, Diagnosis and management) |
| Chung, S. et al. [46] | 2025.08 | US | 106 residents and medical students | 2 parallel arms  (Machine learning dashboard with GutGPT, Machine learning dashboard without GutGPT) | Clinical decision support in acute upper gastrointestinal bleeding management | Residents and medical students | GutGPT (powered by GPT-3.5-turbo-16k or GPT-4-1106-preview) (temperature 0.8, top-p 0.5, maximum token limit 500) | Behavioral Intention  Effort Expectancy  Performance Expectancy  Social Influence  Facilitating Conditions  Trust  Perceived Risk  Perceived Benefit  Clinical decision accuracy  Interaction log patterns |
| Koegel, L. K. et al. [47] | 2025.02 | US | 30 patients | 2 parallel arms  (Autistic patients with Noora, Waitlist) | To improve empathetic statements in autistic adolescents and adults | Autistic adolescents and adults | Noora (powered by GPT-4-0613) | Participants’ empathetic response rate within the AI program  Reliability of the human conversation sample (Generalization)  Reliability of AI program feedback  Confidence survey Acceptability/Satisfaction survey |
| Rollwage, M. et al. [48] | 2026.03 | UK | 227 individuals seeking mental well-being assistance | 3 parallel arms  (LLM with cognitive layer architecture, Standalone LLM, Human clinician) | To deliver high-quality cognitive-behavioral therapy interactions | Human participants who had a relevant mental well-being problem such as low mood or motivation, worry or low self-confidence | GPT-4o-2024-08-06, Claude-3-5-sonnet-20241022, Gemini-1.5-pro, Llama-3-70b  (consistent configuration parameters of temperature 0.7) | Clinical Therapy Performance Scale (CTRS)  Working Alliance Inventory (WAI-SR)  Interpersonal skills  Session structure  Clinical rationale  Potential for patient harm  Procedural completeness  Perceived humanness  Clinical trustworthiness  Appropriateness of topic for a therapeutic conversation  Belief that the evaluated therapist was human  User-reported mood  Trust in AI for mental health support  Perception of whether they were speaking with a human or AI  How human-like interaction felt  Perceived speed of the interaction  Open-ended feedback |
| Li, S. et al. [49] | 2026.01 | China | 2,113 community residents | 2 parallel arms  (Consultation with preparatory e-learning, Consultation only) | To support and enhance patients' health awareness and patient agency in primary care, particularly in resource-limited or underserved settings | Community residents who demonstrate a need for health consultation or express a willingness to engage in LLM-based health consultation | P&P Care (powered by GPT-4o mini) | Objective awareness of health needs  Attention, integrity, listenability, conciseness and empathy  Self-reported awareness of health needs, satisfaction, usefulness, user-friendliness, acceptability and recommendation |
| Liu, Z. et al. [50] | 2026.02 | China | 2,062 patients | 2 parallel arms  (Radiology healthcare providers + Radiology Examination Chatbot, Only radiology healthcare providers) | To optimize clinical interaction between cancer patients and radiology healthcare providers, thereby effectively providing emotional support for cancer patients and reducing burnout | Cancer patients | Radiology Examination Chatbot (powered by fine-tuned DeepSeek R1) | Dialogue quality (empathy, frustration, emotional regulation, factuality, integrity, and satisfaction)  Burnout (exhaustion, depersonalization, and personal achievement)  Image quality of CT/MRI |
| O’Sullivan, J. W. et al. [51] | 2026.02 | US | 9 physicians | 2 parallel arms  (AI assistance, Without AI assistance) | To augment clinical decision-making | Physicians | AMIE (powered by Gemini 2.0 Flash) | Subspecialist preference  Subspecialist individual evaluation  General cardiologist post-assessment  Raw imaging discrepancies |
| Li, J. et al. [52] | 2026.01 | China | 261 patients | 2 parallel arms  (LLM agent, Traditional communication) | To deliver real time, context aware postoperative support after orthopedic surgery | Patients undergoing sports medicine procedures or joint replacement surgeries | WeChat-based LLMs agent (powered by GPT-4-1106) | Generalized Anxiety Disorder (GAD-7)  Functional recovery (e.g., IKDC, FJS)  Health-related quality of life (SF-36)  Patient adherence  Response quality of both the AI and doctor  Patient satisfaction, expectation, and disease-related knowledge |
| Yang, X. et al. [53] | 2026.01 | China | 120 cancer survivors | 3 parallel arms  (AI-enhanced expressive writing, Clinician-guided expressive writing, Usual care) | For reducing depression and anxiety in cancer survivors | Clinically stable cancer survivors without relapse after primary treatment | Therapeutic chatbot (powered by Gemini 1.5 Pro) | Patient Health Questionnaire-9 (PHQ-9)  Generalized Anxiety Disorder (GAD-7)  Fear of Progression Questionnaire-Short Form (FoP-Q-SF)  Connor-Davidson Resilience Scale (CD-RISC-10)  Helpfulness of the writing, quality of feedback, and overall satisfaction  Open-ended questions |
| Tao, X. et al. [54] | 2026.01 | China | 2,069 patients or care partners | 3 parallel arms  (PreA-only, PreA-human, Standard physician only care) | To perform the general medical consultations for history-taking, preliminary diagnoses, and test ordering that would normally be performed by primary care providers and to generate referral reports for specialists | Both the patients (or their caregivers) and the specialist physicians | PreA (powered by fine-tuned GPT-4.0 mini) | Consultation duration  Physician-rated care coordination  Patient-rated ease of communication  Physician workload  Patient-reported experiences of physician attentiveness, satisfaction, interpersonal regard and future acceptability  Physician-reported assessments of PreA’s utility, ease of communication, and workload relief  Clinical decision-making patterns |
| Koç, U. et al. [55] | 2026.01 | Türkiye | 122 patients or healthy people | 2 parallel arms  (ChatGPT-generated informed consent, Standard informed consent) | Generating informed consent forms in interventional radiology | Not reported | ChatGPT-4o (May 27, 2024) | Difference in overall comprehension scores of the knowledge test between groups  Test–retest assessment and attitudinal responses |
| Navarese, E. P. et al. [56] | 2026.03 | UK | 60 Patients | 2 parallel arms  (Virtual assistant guided care, Standard of care) | To optimize guideline-directed medical therapy in heart failure (HF) with reduced ejection fraction | Nonphysician staff | SIRIO-HF VA (powered by GPT-4 with retrieval augmented generation) | Feasibility, acceptability to users, safety, and effectiveness  NYHA class  N-terminal pro–B-type natriuretic peptide (NT-proBNP)  Number of HF-related hospitalizations  Echocardiographic left ventricular assessment  Patient reported quality of life by the Kansas City Cardiomyopathy Questionnaire (KCCQ-12)  Agreement between the virtual assistant and the clinician. |
| Nguyen, Q. C. et al. [57] | 2026.01 | US | 84 mothers | 2 parallel arms  (Rosie mobile app, Children’s board book) | To deliver evidence-based health information and prevent avoidable adverse health outcomes in mothers and newborns | Pregnant and postpartum women | Rosie (powered by Llama 3.1) | Response quality  Clarity  Usefulness  Feature engagement  Satisfaction  User interaction  User ratings of response |
| Sun, Y. et al. [58] | 2026.03 | China | 274 patients | 3 parallel arms  (Physician voice cloning education, Patient’s own voice cloning education, Traditional education) | To deliver personalized, scalable, and interactive inpatient health education | Medical professionals | Resemble.ai | Education content compliance rate  Knowledge mastery  Education satisfaction  Treatment adherence  Quality of life (SF-36)  Anxiety and depression (HADS) |
| Ouyang, H. et al. [59] | 2026.01 | China | 127 caregivers | 2 parallel arms  (LLM assisted education, Standard web-based educational materials) | For health education on developmental dysplasia of the hip | Caregivers of children aged 0-14 years | Not reported | eHealth Literacy Scale (eHEALS)  Developmental dysplasia of the hip knowledge test (DDH-KT)  Health Risk Perception Scale (HRPS)  Information Self-Efficacy Scale (ISES)  Perceived Usefulness Scale (PUS)  Health Information-Seeking Behavior Scale (HISBS) |
| Everett, S. S. et al. [60] | 2026.03 | US | 70 Physicians | 2 parallel arms  (AI as second opinion, AI as first opinion) | For collaborative diagnostic reasoning (DDx) | Physicians | Custom GPT-4 (powered by GPT-4) | Total graded vignette score  Difference in total score compared to clinicians using conventional resources  Time spent on each case  Change in score of the evidence section of the vignette  Change in score of the clinically actionable decisions |
| Rahrisch, A. et al. [61] | 2026.02 | Switzerland | 30 physicians | 2 crossover arms  (AI-supported documentation, Manual documentation) | To automate documentation during preanesthetic consultations | Physicians | Issac (powered by GPT-4) | Total time of the preanesthetic consultation  Visual attention during documentation  Human–computer interaction  Subjective workload  Documentation quality  Self-assessed quality of report and patient interaction  Preferred support for documentation and workflow |
| Yıldız, H.A. [62] | 2026.01 | Türkiye | 100 patients | 5 parallel arms  (ChatGPT, Gemini, Perplexity, Copilot, Human doctors) | To provide urinary stone disease consultations | Patients newly diagnosed with urinary stone disease | ChatGPT-4o, Gemini 1.5, Perplexity Pro, Copilot version 1.25011.96.0 | Client Satisfaction Questionnaire-8 (CSQ-8)  Consultation duration  Number of patient questions |
| Camathias, C. et al. [63] | 2026.02 | Switzerland | 20 pediatric patients | 2 parallel arms  (ChatGPT-generated, Human-generated) | Generating medical history summaries in pediatric orthopaedic practice | Physicians | ChatGPT-4o (version as of January 2025) | Temporal consistency  Spatial consistency  Accident description  Symptom accuracy  Symptom specificity  Previous interventions  Writing style  Overall impression |
| Holderried, F. et al. [64] | 2026.02 | German | 48 standardized patients | 2 crossover arms  (GPT-4–generated patient letter, Standard hospital discharge letter) | Transforming discharge letters into patient letters | Not reported | GPT-4 | Proportion of learning objectives fully, partially, or not reported  Content field (medication, organization, prevention of complications, lifestyle/disease management)  Bloom taxonomy level |
| Chen, A. H. et al. [65] | 2025.11 | US | 2,000 general population | 2 crossover arms  (AI generated plain language report, Original report) | Simplification of radiology reports into plain language | Not reported | GPT-4 Turbo (November 28th, 2023) | Comprehension  Whether participants could accurately determine the appropriate follow-up timeline based on the findings.  Self-reported confidence in comprehension accuracy  Perceived difficulty of comprehension  Specific aspects of perceived difficulty  Level of perceived anxiety |
| McFadyen, J. et al. [66] | 2026.01 | UK | 531 Patietns | 2 parallel arms  (Limbic care, Digital workbook) | Increasing engagement and reducing anxiety, depression, and sleep disturbances with cognitive-behavioral therapy | Patients with anxiety and/or depression symptoms above threshold scores on widely validated screening measures | Limbic Care | Engagement with therapeutic materials  Anxiety and depression symptom severity (GAD-7, PHQ-9)  Adverse events reported by participants  Work and Social Adjustment Scale (WSAS)  Mini Sleep Questionnaire (MSQ)  User experience  Beliefs and attitudes towards AI & psychotherapy |
| Bean, A. M. et al. [67] | 2026.02 | UK | 615 general population | 4 parallel arms  (GPT-4o, Llama-3, Command R+, Online assistance without LLM) | To assist members of the public in identifying underlying conditions and choosing a course of action | General population | GPT-4o-2024-05-13, Meta-Llama-3-70B-Instruct, Command-r-plus-08-2024  (temperature 0.8, top-p 0.9, top-k 5, maximum token limit 256 except 1506 for Command R+) | Disposition accuracy  Identifying underlying conditions  Rates of over- and underestimation of severity  Self-reported confidence  Reliance on different sources of information  Trust in LLMs for general and medical purposes  Likelihoods that they would recommend LLMs to their family and friends  Precision |
| Mutlu Sütcüoğlu, B., Öztop, M.M. & Hansu, K. [68] | 2026.04 | Türkiye | 300 pregnant women | 2 parallel arms  (ChatGPT and consultation, Only consultation) | In reducing preoperative anxiety in women undergoing elective cesarean sections | Pregnant women scheduled for cesarean section | ChatGPT | State-Trait Anxiety Inventory (STAI) I & II  Edinburgh Postnatal Depression Scale (EPDS)  Visual pain scores (VPS)  Accuracy and reliability |
| Heinz, M.V. et al. [69] | 2025.03 | US | 203 patients | 2 parallel arms  (Interacting with Therabot, Waitlist) | Treatment for major depressive disorder, generalized anxiety disorder, or clinically high-risk feeding and eating disorders | Patients with major depressive disorder, generalized anxiety disorder, or clinically high-risk feeding and eating disorders | Therabot, (powered by fine-tuned Falcon-7B and LLaMA-2-70B) | PHQ-9  GAD-Q-IV  Weight Concerns Sclae  Working Alliance Inventory-Short Revised (WAI-SR)  Engagement  Satisfaction |
| Song, Y. et al. [70] | 2025.08 | China | 114 patients | 2 parallel arms  (Receiving M-AI psychological intervention, Standard discharge care) | To improve post-discharge care for patients with cardiovascular disease in resource-limited settings. | Patients with cardiovascular disease in resource-limited settings | Medical Generative AI Assistant for Post-Discharge Intervention in Cardiovascular Disease (M-AI) | State–Trait Anxiety Inventory (STAI)  30-day readmission rate |
| Qazi, I.A. et al. [71] | 2026.02 | Pakistan | 58 physicians | 2 parallel arms  (Physicians plus LLM, Physicians plus conventional resources) | To improve diagnostic reasoning | Physicians | ChatGPT 4o | Composite diagnostic reasoning score  Time Spent on Diagnosis  Final diagnosis accuracy |
| Etagiuri, A. et al. [72] | 2026.04 | Canada | 279 Patients | 2 parallel arms  (AI scribe, Conventional) | Generating summaries using either standard or surgeon-specific templates. | Physicians | Heidi AI Medical Scribe | Surgeon documentation time  Administrative processing time  Any technical failures of the software  Documentation accuracy  Patient satisfaction |
| Shin, H. S. et al. [73] | 2025.09 | US | 23 physicians | 2 parallel arms  (DAX Copilot, Documentation as usual) | To produce provider note sections from recordings of patient-provider encounters | Pediatric physician | DAX Copilot | Net EHR Experience Score  Experiencing burnout  Pajama time  Time in notes per appointment  Outpatient adjusted work related value units per encounter |
| Kakaday, R. et al. [74] | 2025.07 | US | 50 clinicians | 2 parallel arms  (DAX Copilot, Traditional documentation) | To improve clinical documentation efficiency | Primary care clinician | DAX Copilot | Documentation time  Documentation length  Documentation composition by DAX |

1.	Cheng ST, Ng PHF. Intervention Without Borders – an Automated Self-Guided AI-Enhanced Psychoeducation Intervention for Dementia Caregivers: Parallel-Group Randomized Waitlist-Controlled Trial. American Journal of Geriatric Psychiatry. 2026. doi: 10.1016/j.jagp.2026.01.001.

2.	Cheng S-T, Ng PHF. Multiple Therapeutic Mechanisms of an Automated Self-Guided AI-Enhanced Psychoeducation Intervention for Dementia Family Caregivers: Randomized Waitlist-Controlled Trial. The American Journal of Geriatric Psychiatry. 2026 2026/02/23/. doi: https://doi.org/10.1016/j.jagp.2026.02.003.

3.	Zhao H, Bai Y, Chen L, Ma J, Lei Y, Sun T, et al. Generative AI-based low-dose digital subtraction angiography for intra-operative radiation dose reduction: a randomized controlled trial. Nature Medicine. 2026;32(1):288–96. doi: 10.1038/s41591-025-04042-6.

4.	Cao Y, Lu W, Shi R, Liu F, Liu S, Xu X, et al. Performance of popular large language models in glaucoma patient education: A randomized controlled study. Adv Ophthalmol Pract Res. 2025 May–Jun;5(2):88–94. PMID: 40162329. doi: 10.1016/j.aopr.2024.12.002.

5.	Goh E, Gallo R, Hom J, Strong E, Weng Y, Kerman H, et al. Large Language Model Influence on Diagnostic Reasoning: A Randomized Clinical Trial. JAMA Netw Open. 2024 Oct 1;7(10):e2440969. PMID: 39466245. doi: 10.1001/jamanetworkopen.2024.40969.

6.	Wan P, Huang Z, Tang W, Nie Y, Pei D, Deng S, et al. Outpatient reception via collaboration between nurses and a large language model: a randomized controlled trial. Nat Med. 2024 Oct;30(10):2878–85. PMID: 39009780. doi: 10.1038/s41591-024-03148-7.

7.	Baker HP, Dwyer E, Kalidoss S, Hynes K, Wolf J, Strelzow JA. ChatGPT's Ability to Assist with Clinical Documentation: A Randomized Controlled Trial. JAAOS - Journal of the American Academy of Orthopaedic Surgeons. 2024;32(3):123–9. PMID: 00124635-202402010-00007. doi: 10.5435/jaaos-d-23-00474.

8.	McDuff D, Schaekermann M, Tu T, Palepu A, Wang A, Garrison J, et al. Towards accurate differential diagnosis with large language models. Nature. 2025 Apr 9. PMID: 40205049. doi: 10.1038/s41586-025-08869-4.

9.	Gorenshtein A, Weisblat Y, Khateb M, Kenan G, Tsirkin I, Fayn G, et al. AI-Based EMG Reporting: A Randomized Controlled Trial. Journal of Neurology. 2025;272(9). doi: 10.1007/s00415-025-13261-3.

10.	Wu Y, Chen X, Zhang W, Liu S, Sum WMR, Wu X, et al. ChatMyopia: An AI agent for myopia-related consultation in primary eye care settings. iScience. 2025 Nov 21;28(11):113768. PMID: 41244561. doi: 10.1016/j.isci.2025.113768.

11.	Ke YH, Yang Ong BS, Jin L, Sim JXL, Chan CH, Soh CR, et al. Clinical and economic impact of a large language model in perioperative medicine: a randomized crossover trial. npj Digital Medicine. 2025;8(1). doi: 10.1038/s41746-025-01858-x.

12.	Tailor PD, D'Souza HS, Castillejo Becerra CM, Dahl HM, Patel NR, Kaplan TM, et al. Evaluation of AI Summaries on Interdisciplinary Understanding of Ophthalmology Notes. JAMA Ophthalmology. 2025;143(5):410–9. doi: 10.1001/jamaophthalmol.2025.0351.

13.	Ye X, Shan X, Tu Y, Zhang Y. Examining the Efficacy of Large Language Models for Mitigating Depression and Anxiety Among Chinese Students: A Randomized Controlled Trial. Computers, informatics, nursing : CIN. 2025;43(9). doi: 10.1097/CIN.0000000000001349.

14.	Sabour S, Zhang W, Xiao X, Zhang Y, Zheng Y, Wen J, et al. A chatbot for mental health support: exploring the impact of Emohaa on reducing mental distress in China. Frontiers in Digital Health. 2023;5. doi: 10.3389/fdgth.2023.1133987.

15.	F A, ÖT Y, AH A, B M, CH B. Comparison of artificial intelligence-assisted informed consent obtained before coronary angiography with the conventional method: Medical competence and ethical assessment. Digital health. 2023 2023–1;9:20552076231218141. PMID: rayyan-512511070. doi: doi:10.1177/20552076231218141.

16.	Tai-Seale M, Baxter SL, Vaida F, Walker A, Sitapati AM, Osborne C, et al. AI-Generated Draft Replies Integrated Into Health Records and Physicians' Electronic Communication. JAMA Netw Open. 2024 Apr 1;7(4):e246565. PMID: 38619840. doi: 10.1001/jamanetworkopen.2024.6565.

17.	Yahagi M, Hiruta R, Miyauchi C, Tanaka S, Taguchi A, Yaguchi Y. Comparison of Conventional Anesthesia Nurse Education and an Artificial Intelligence Chatbot (ChatGPT) Intervention on Preoperative Anxiety: A Randomized Controlled Trial. J Perianesth Nurs. 2024 Oct;39(5):767–71. PMID: 38520470. doi: 10.1016/j.jopan.2023.12.005.

18.	Chung D, Sidhom K, Dhillon H, Bal DS, Fidel MG, Jawanda G, et al. Real-world utility of ChatGPT in pre-vasectomy counselling, a safe and efficient practice: a prospective single-centre clinical study. World J Urol. 2024 Dec 14;43(1):32. PMID: 39673635. doi: 10.1007/s00345-024-05385-4.

19.	Ebnali Harari R, Altaweel A, Ahram T, Keehner M, Shokoohi H. A randomized controlled trial on evaluating clinician-supervised generative AI for decision support. International Journal of Medical Informatics. 2025;195. doi: 10.1016/j.ijmedinf.2024.105701.

20.	Hou Z, Wu Z, Qu Z, Gong L, Peng H, Jit M, et al. A vaccine chatbot intervention for parents to improve HPV vaccination uptake among middle school girls: a cluster randomized trial. Nature Medicine. 2025;31(6):1855–62. doi: 10.1038/s41591-025-03618-6.

21.	Lukac PJ, Turner W, Vangala S, Chin AT, Khalili J, Shih YT, et al. Ambient AI Scribes in Clinical Practice: A Randomized Trial. Nejm ai. 2025 Dec;2(12). PMID: 41497288. doi: 10.1056/aioa2501000.

22.	Wu Y, Qian B, Li T, Qin Y, Guan Z, Chen T, et al. An eyecare foundation model for clinical assistance: a randomized controlled trial. Nature Medicine. 2025;31(10):3404–13. doi: 10.1038/s41591-025-03900-7.

23.	Berzolla E, Gosnell GG, Chen L, Vonck C, Alaia E, Meislin R. Artificial Intelligence Large Language Models Improve Patient Comprehension of Radiologist Magnetic Resonance Imaging Reports. Arthroscopy - Journal of Arthroscopic and Related Surgery. 2025;41(11):4607–14.e4. doi: 10.1016/j.arthro.2025.04.033.

24.	Kerimoglu Yildiz G, Turk Delibalta R, Coktay Z. Artificial intelligence-assisted chatbot: impact on breastfeeding outcomes and maternal anxiety. BMC Pregnancy and Childbirth. 2025;25(1). doi: 10.1186/s12884-025-07753-3.

25.	Aydemir U. ChatGPT-Delivered Physical Activity Intervention for Children With Autism Spectrum Disorder: Pre-Post Feasibility Study. JMIR Hum Factors. 2025 Jun 11;12:e71119. PMID: 40498657. doi: 10.2196/71119.

26.	Gan W, Ouyang J, She G, Xue Z, Zhu L, Lin A, et al. ChatGPT's role in alleviating anxiety in total knee arthroplasty consent process: a randomized controlled trial pilot study. Int J Surg. 2025 Mar 1;111(3):2546–57. PMID: 39903546. doi: 10.1097/JS9.0000000000002223.

27.	Chen C, Lam KT, Yip KM, So HK, Lum TYS, Wong ICK, et al. Comparison of an AI Chatbot With a Nurse Hotline in Reducing Anxiety and Depression Levels in the General Population: Pilot Randomized Controlled Trial. JMIR Hum Factors. 2025 Mar 6;12:e65785. PMID: 40048637. doi: 10.2196/65785.

28.	Weuthen FA, Otte N, Krabbe H, Kraus T, Krabbe J. Comparison of ChatGPT and Internet Research for Clinical Research and Decision-Making in Occupational Medicine: Randomized Controlled Trial. JMIR Form Res. 2025 May 20;9:e63857. PMID: 40393042. doi: 10.2196/63857.

29.	Zhang H, Wang X, Luo H, Zeng W, Hong X, Feng J, et al. Comparison of preoperative education by artificial intelligence versus traditional physicians in perioperative management of urolithiasis surgery: a prospective single-blind randomized controlled trial conducted in China. Front Med (Lausanne). 2025;12:1543630. PMID: 40636362. doi: 10.3389/fmed.2025.1543630.

30.	Akdogan O, Uyar GC, Yesilbas E, Baskurt K, Malkoc NA, Ozdemir N, et al. Effect of a ChatGPT-based digital counseling intervention on anxiety and depression in patients with cancer: A prospective, randomized trial. Eur J Cancer. 2025 May 15;221:115408. PMID: 40215593. doi: 10.1016/j.ejca.2025.115408.

31.	Zhao Y, Qian W, Chen Y, Wu D, Luo Y, Gao C, et al. Effect of an AI agent trained on a large language model (LLM) as an intervention for depression and anxiety symptoms in young adults: A 28-day randomized controlled trial. Applied psychology Health and well-being. 2025;17(5):e70067. doi: 10.1111/aphw.70067.

32.	Lai HL, Chen CI, Liaw GH, Lu LC, Huang CY. Effectiveness of a Chatbot-Based Internet-Support-Coping-Caring Intervention in Improving Sleep Quality and Work-Related Outcomes Among Nurses in Taiwan During the COVID-19 Pandemic: A Pilot Study. The journal of nursing research : JNR. 2025;33(5):e411. doi: 10.1097/jnr.0000000000000691.

33.	Anand E, Ghersin I, Lingam G, Devlin K, Pelly T, Singer D, et al. Enhancing Patient Understanding of Perianal Fistula MRI Findings Using ChatGPT: A Randomized, Single Centre Study. Diagnostics. 2026;16(1). doi: 10.3390/diagnostics16010072.

34.	Islam S. Evaluating the impact of AI-generated educational content on patient understanding and anxiety in endodontics and restorative dentistry: a comparative study. BMC oral health. 2025;25(1):689. doi: 10.1186/s12903-025-06069-0.

35.	Busch F, Kaibel L, Nguyen H, Lemke T, Ziegelmayer S, Graf M, et al. Evaluation of a Retrieval-Augmented Generation-Powered Chatbot for Pre-CT Informed Consent: a Prospective Comparative Study. J Imaging Inform Med. 2025 Dec;38(6):4312–23. PMID: 40119020. doi: 10.1007/s10278-025-01483-w.

36.	Donner S, Knauer P, Kienzle A, Dinneen J, Burger J, Perka C, et al. Exploring Patient Perspectives, Engagement, and Output Quality in Doctor-Supervised Use of Artificial Intelligence During Informed Consent Consultation With ChatGPT and Retrieval Augmented Generation (RAG): Quantitative Exploratory Study. Journal of Medical Internet Research. 2025;27(1). doi: 10.2196/73717.

37.	Goh E, Gallo RJ, Strong E, Weng Y, Kerman H, Freed JA, et al. GPT-4 assistance for improvement of physician performance on patient care tasks: a randomized controlled trial. Nature Medicine. 2025;31(4):1233–8. doi: 10.1038/s41591-024-03456-y.

38.	Stephan D, Bertsch AS, Schumacher S, Puladi B, Burwinkel M, Al-Nawas B, et al. Improving Patient Communication by Simplifying AI-Generated Dental Radiology Reports With ChatGPT: Comparative Study. Journal of Medical Internet Research. 2025;27. doi: 10.2196/73337.

39.	Allen JW, Hannikainen IR, Savulescu J, Wilkinson D, Earp BD. Is Consent-GPT valid? Public attitudes to generative AI use in surgical consent. AI & SOCIETY. 2026 2026/03/01;41(3):2637–55. doi: 10.1007/s00146-025-02644-9.

40.	Samardzic D, Curkovic J, Okmazic D, Glumac S, Vrdoljak J, Skara Kolega M, et al. Patients Prefer Human Empathy, but Not Always Human Wording: A Single-Blind Within-Subject Trial of GPT-Generated vs. Clinician Discharge Texts in Emergency Ophthalmology. Clinics and Practice. 2025;15(11). doi: 10.3390/clinpract15110208.

41.	Goh E, Bunning B, Khoong EC, Gallo RJ, Milstein A, Centola D, et al. Physician clinical decision modification and bias assessment in a randomized controlled trial of AI assistance. Commun Med (Lond). 2025 Mar 4;5(1):59. PMID: 40038550. doi: 10.1038/s43856-025-00781-2.

42.	Gupta A, Singh S, Malhotra H, Pruthi H, Sharma A, Garg AK, et al. Provision of Radiology Reports Simplified with Large Language Models to Patients with Cancer: Impact on Patient Satisfaction. JCO Clinical Cancer Informatics. 2025;9. doi: 10.1200/CCI-24-00166.

43.	Indrayanti I, Salsabila AK, Amrita V, Alhaddad MM, Saskia AB, Ramadhani DP. PsyBot: A randomized controlled trial of WhatsApp-based psychological first aid to reduce loneliness among 18–22-year-old students in Yogyakarta, Indonesia. SSM - Mental Health. 2025;8. doi: 10.1016/j.ssmmh.2025.100504.

44.	Campellone TR, Flom M, Montgomery RM, Bullard L, Pirner MC, Pavez A, et al. Safety and User Experience of a Generative Artificial Intelligence Digital Mental Health Intervention: Exploratory Randomized Controlled Trial. Journal of Medical Internet Research. 2025;27. doi: 10.2196/67365.

45.	Tu T, Schaekermann M, Palepu A, Saab K, Freyberg J, Tanno R, et al. Towards conversational diagnostic artificial intelligence. Nature. 2025 Apr 9. PMID: 40205050. doi: 10.1038/s41586-025-08866-7.

46.	Chung S, Giuffrè M, Rajashekar N, Pu Y, Shin YE, Kresevic S, et al. Usability and adoption in a randomized trial of GutGPT a GenAI tool for gastrointestinal bleeding. npj Digital Medicine. 2025;8(1). doi: 10.1038/s41746-025-01896-5.

47.	Koegel LK, Ponder E, Bruzzese T, Wang M, Semnani SJ, Chi N, et al. Using Artificial Intelligence to Improve Empathetic Statements in Autistic Adolescents and Adults: A Randomized Clinical Trial. J Autism Dev Disord. 2025 Feb 15. PMID: 39954218. doi: 10.1007/s10803-025-06734-x.

48.	Rollwage M, McFadyen J, Juchems K, Balogh A, Pisupati S, Mircea MT, et al. A cognitive layer architecture to support large-language model performance in psychotherapy interactions. Nature Medicine. 2026. doi: 10.1038/s41591-026-04278-w.

49.	Li S, Li Y, Zhou S, Tao X, Yu C, Shen M, et al. A community-codesigned LLM-powered chatbot for primary care: a randomized controlled trial. Nat Health. 2026;1(2):238–50. PMID: 41659358. doi: 10.1038/s44360-025-00021-w.

50.	Liu Z, Orouji F, Yang F, Shi Z, Liang H, Yu B, et al. A fine-tuned large language model chatbot for multi-scenario radiology cancer care: randomized controlled trial on interaction optimization, emotional support, and provider burnout reduction. Journal of Translational Medicine. 2026;24(1). doi: 10.1186/s12967-026-07738-6.

51.	O’Sullivan JW, Palepu A, Saab K, Weng WH, Amponsah DK, Cheng E, et al. A large language model for complex cardiology care. Nature Medicine. 2026;32(2):616–23. doi: 10.1038/s41591-025-04190-9.

52.	Li J, Zhang Y, Zhang Z, Zhou Y, Gao Y, Li X, et al. A randomized controlled trial of a WeChat-based artificial intelligence agent for postoperative care in orthopedic patients. npj Digital Medicine. 2026;9(1). doi: 10.1038/s41746-025-02269-8.

53.	Yang X, Huo Y, Wang Y, Li M, Zhang R, Liu L, et al. AI-Enhanced Versus Clinician-Guided Expressive Writing for Improving Psychological Well-Being in Cancer Survivors: A Randomized Controlled Trial. Psycho-Oncology. 2026;35(1). doi: 10.1002/pon.70386.

54.	Tao X, Zhou S, Ding K, Li S, Li Y, Wu B, et al. An LLM chatbot to facilitate primary-to-specialist care transitions: a randomized controlled trial. Nature Medicine. 2026;32(3):934–42. doi: 10.1038/s41591-025-04176-7.

55.	Koç U, Güneş YC, Çolakoğlu MN, Coşkun N, Dere V, Özdemir M, et al. ChatGPT-generated informed consent forms in interventional radiology: A randomized controlled evaluation of comprehension, attitudinal responses, and readability. European Journal of Radiology. 2026;195. doi: 10.1016/j.ejrad.2025.112624.

56.	Navarese EP, Leader JH, Markides RIL, Koolaji S, Kereiakes DJ, Kubica J, et al. Design and Architecture of a Generative-AI-Supported, Nonphysician-Delivered Model for GDMT Optimization in HFrEF: The ASSIST-HF Trial. JACC: Advances. 2026 2026/03/01/;5(3):102588. doi: https://doi.org/10.1016/j.jacadv.2026.102588.

57.	Nguyen QC, Norell EM, Mane H, Yue X, Srikanth NP, Peters CJ, et al. Developing an artificial intelligence-powered question-and-answer chatbot with English-Spanish capabilities for new mothers. JAMIA Open. 2026;9(1). doi: 10.1093/jamiaopen/ooaf169.

58.	Sun Y, Xu S, Jin H, Han X, Jin K, Zhang Y, et al. Effectiveness of Al-Assisted Patient Health Education Using Voice Cloning and ChatGPT: Prospective Randomized Controlled Trial. Journal of medical Internet research. 2026;28:e81387. doi: 10.2196/81387.

59.	Ouyang H, Lin G, Li Y, Yao Z, Li Y, Yan H, et al. Evaluating and Validating Large Language Models for Health Education on Developmental Dysplasia of the Hip: 2-Phase Study With Expert Ratings and a Pilot Randomized Controlled Trial. Journal of Medical Internet Research. 2026;28. doi: 10.2196/73326.

60.	Everett SS, Bunning BJ, Jain P, Lopez I, Agarwal A, Desai M, et al. From tool to teammate in a randomized controlled trial of clinician-AI collaborative workflows for diagnosis. npj Digital Medicine. 2026 2026/03/18;9(1):409. doi: 10.1038/s41746-026-02545-1.

61.	Rahrisch A, Braun J, Ganter MT, Roche TR, Spahn DR, Tscholl DW, et al. Generative AI in preanesthetic consultations: Effects on efficiency, documentation workload, quality, and physician–patient interaction: A simulation trial. Journal of Clinical Anesthesia. 2026;110. doi: 10.1016/j.jclinane.2026.112163.

62.	Yıldız HA. Human Doctors or AI? Evaluating Patient Satisfaction in Urinary Stone Disease Consultations. Journal of Surgical Research. 2026;317:181–8. doi: 10.1016/j.jss.2025.11.026.

63.	Camathias C, Papp K, Betschart P, Speth B, Valderrabano V, Ammann E. Human medical documentation significantly outperforms ChatGPT-4o in critical clinical dimensions: A blinded comparative assessment in paediatric orthopaedics. Knee surgery, sports traumatology, arthroscopy : official journal of the ESSKA. 2026. doi: 10.1002/ksa.70357.

64.	Holderried F, Sonanini A, Stegemann–Philipps C, Herrmann–Werner A, Spitzer P, Guthoff M, et al. Impact of GPT-4–Generated Discharge Letters on Patients’ Medical Comprehension: Prospective Crossover Study. Journal of Medical Internet Research. 2026;28. doi: 10.2196/81243.

65.	Chen AH, Rudin RS, Levine DM, Mehrotra A. Improving patient understanding of radiology reports using generative artificial intelligence: a vignette study of 2000 US adults. Journal of the American Medical Informatics Association. 2026;33(2):326–35. doi: 10.1093/jamia/ocaf187.

66.	McFadyen J, Habicht J, Dina L-M, Harper R, Hauser TU, Rollwage M. Increasing engagement with cognitive-behavioral therapy (CBT) using generative AI: a randomized controlled trial (RCT). Communications Medicine. 2026 2026/01/15;6(1):129. doi: 10.1038/s43856-025-01321-8.

67.	Bean AM, Payne RE, Parsons G, Kirk HR, Ciro J, Mosquera-Gómez R, et al. Reliability of LLMs as medical assistants for the general public: a randomized preregistered study. Nature Medicine. 2026;32(2):609–15. doi: 10.1038/s41591-025-04074-y.

68.	Mutlu Sütcüoğlu B, Öztop MM, Hansu K. The impact of ChatGPT on preoperative anxiety and postoperative depression in cesarean section: a prospective randomized trial. BMC Pregnancy and Childbirth. 2026 2026/04/01;26(1):517. doi: 10.1186/s12884-026-09011-6.

69.	Heinz MV, Mackin DM, Trudeau BM, Bhattacharya S, Wang Y, Banta HA, et al. Randomized Trial of a Generative AI Chatbot for Mental Health Treatment. NEJM AI. 2025;2(4):AIoa2400802. doi: doi:10.1056/AIoa2400802.

70.	Song Y, Yan T, Jia F, Chen L, Li H. Developing Generative AI for Value Co-Creation: An Intervention-Based Randomized Field Experiment in a Healthcare Context. Journal of Operations Management. 2026;72(2):197–232. doi: https://doi.org/10.1002/joom.70012.

71.	Qazi IA, Ali A, Khawaja AU, Akhtar MJ, Sheikh AZ, Alizai MH. Large language model diagnostic assistance for physicians in a lower-middle-income country: a randomized controlled trial. Nature Health. 2026 2026/02/01;1(2):198–205. doi: 10.1038/s44360-025-00007-8.

72.	Etagiuri A, Chaudhry D, van der Merwe J, Nickol M, van der Merwe JM. Evaluating the Impact of Artificial Intelligence Scribes on Clinical Workflow and Documentation Quality: A Randomized Controlled Trial. The Journal of Arthroplasty. 2026 2026/04/02/. doi: https://doi.org/10.1016/j.arth.2026.03.069.

73.	Shin HS, Williams H, Braykov N, Jahan A, Meller J, Orenstein EW. The Influence of Artificial Intelligence Scribes on Clinician Experience and Efficiency among Pediatric Subspecialists: A Rapid, Randomized Quality Improvement Trial. Applied clinical informatics. 2025;16(4):1041–52. doi: 10.1055/a-2657-8087.

74.	Kakaday R, Herrera EZ, Coskey O, Hertel AW, Kaiser P. The STREAMLINE Pilot Study on Time Reduction and Efficiency in AI-Mediated Logging for Improved Note-Taking Experience. Appl Clin Inform. 2025 May;16(3):614–21. PMID: 40097146. doi: 10.1055/a-2559-5791.
