# Supplementary material for: Reporting Gaps and Challenges in Randomized Trials of Generative AI for Medical Use: Insights From a Systematic Review
Source: J Med Internet Res. 2026 Oct 28;28:e94543. doi: 10.2196/94543 (PMC13718382; doi:10.2196/94543)
Supplement: Supplementary file 8 [file jmir-v28-e94543-s006.docx]

Table S7. Sensitivity analysis of item-level adherence using proportional scoring of signaling questions

| CONSORT-AI item | Signaling questions, n | Fully reported (all-or-none),  n/N (%)^a^ | Mean proportion of signaling questions satisfied (SD) (%)^b^ |
| --- | --- | --- | --- |
| 1a, b (ⅰ) | 2 | 64/74 (86.5) | 91.9 (21.9) |
| 1a, b (ⅱ) | 2 | 73/74 (98.6) | 98.6 (11.6) |
| 2a (ⅰ) | 2 | 59/74 (79.7) | 89.2 (22.3) |
| 4a (ⅰ) | 1 | 66/74 (89.2) | 89.2 (31.3) |
| 4a (ⅱ) | 4 | 3/73 (4.1) | 19.5 (22.9) |
| 4b | 2 | 21/74 (28.4) | 29.1 (45.3) |
| 5 (ⅰ) | 6 | 17/74 (23.0) | 30.5 (41.6) |
| 5 (ⅱ) | 3 | 9/74 (12.2) | 46.2 (24.4) |
| 5 (ⅲ) | 8 | 0/72 (0.0) | 5.3 (15.9) |
| 5 (ⅳ) | 3 | 28/74 (33.8) | 68.7 (28.8) |
| 5 (ⅴ) | 1 | 70/74 (94.6) | 94.6 (22.8) |
| 5 (ⅵ) | 4 | 35/68 (51.6) | 59.1 (45.7) |
| 19 | 5 | 28/74 (36.5) | 41.8 (41.9) |
| 25 | 1 | 31/74 (41.9) | 41.9 (49.7) |

^a^Reports in which the item was rated not applicable were excluded from the denominator; denominators therefore differ across items.
^b^In this sensitivity analysis, adherence was recalculated for each trial as the number of signaling questions satisfied divided by the number of applicable signaling questions for that item, and the mean across reports is presented. Signaling questions rated as partially satisfied were counted as not satisfied, and those rated not applicable were excluded from the denominator. For items comprising a single signaling question, the two estimates are identical by definition.
