# Supplementary material for: Reporting Gaps and Challenges in Randomized Trials of Generative AI for Medical Use: Insights From a Systematic Review
Source: J Med Internet Res. 2026 Oct 28;28:e94543. doi: 10.2196/94543 (PMC13718382; doi:10.2196/94543)
Supplement: Supplementary file 9 [file jmir-v28-e94543-s007.docx]

Table S8. Subgroup analysis (Region)

| No | CONSORT-AI checklist | North America  n/23 (%) | Asia  n/37 (%) | Europe  n/14 (%) | *p-*value | *q*-value |
| --- | --- | --- | --- | --- | --- | --- |
| 1a, b (ⅰ) | AI intervention stated in title/abstract | 22/23 (96%) | 28/37 (76%) | 14/14 (100%) | 0.031 | 0.199 |
| 1a, b (ⅱ) | Intended use stated in title/abstract | 22/23 (96%) | 37/37 (100%) | 14/14 (100%) | 0.500 | 0.644 |
| 2a (ⅰ) | Intended use and user in clinical pathway in introduction | 18/23 (78%) | 31/37 (84%) | 10/14 (71%) | 0.594 | 0.644 |
| 4a (ⅰ) | Participant-level eligibility criteria | 19/23 (83%) | 37/37 (100%) | 10/14 (71%) | 0.007 | 0.091 |
| 4a (ⅱ) | Input-data-level eligibility criteria | 2/22 (9%) | 0/37 (0%) | 1/14 (7%) | 0.169 | 0.549 |
| 4b | Integration into the trial setting | 8/23 (35%) | 11/37 (30%) | 2/14 (14%) | 0.438 | 0.644 |
| 5 (ⅰ) | Version of the AI algorithm | 7/23 (30%) | 7/37 (19%) | 3/14 (21%) | 0.577 | 0.644 |
| 5 (ⅱ) | Input-data acquisition and selection | 3/23 (13%) | 3/37 (8%) | 3/14 (21%) | 0.455 | 0.644 |
| 5 (ⅲ) | Handling of poor-quality or missing data | 0/23 (0%) | 0/36 (0%) | 0/13 (0%) | – | – |
| 5 (ⅳ) | Human-AI interaction and user expertise | 8/23 (35%) | 14/37 (38%) | 3/14 (21%) | 0.582 | 0.644 |
| 5 (ⅴ) | Output of the AI intervention | 21/23 (91%) | 35/37 (95%) | 14/14 (100%) | 0.700 | 0.700 |
| 5 (ⅵ) | Contribution of output to decision-making | 14/20 (70%) | 16/37 (43%) | 5/11 (45%) | 0.151 | 0.549 |
| 19 | Performance errors and error analysis | 10/23 (43%) | 11/37 (30%) | 6/14 (43%) | 0.560 | 0.644 |
| 25 | Access to the AI intervention or code | 11/23 (48%) | 13/37 (35%) | 7/14 (50%) | 0.523 | 0.644 |

Table S9. Subgroup analysis (Year)

| No | CONSORT-AI checklist | 2023  n/2 (%) | 2024  n/8 (%) | 2025  n/39 (%) | 2026  n/25 (%) | *p-*value | *q*-value |
| --- | --- | --- | --- | --- | --- | --- | --- |
| 1a, b (ⅰ) | AI intervention stated in title/abstract | 1/2 (50%) | 8/8 (100%) | 34/39(87%) | 21/25(84%) | 0.348 | 0.904 |
| 1a, b (ⅱ) | Intended use stated in title/abstract | 2/2 (100%) | 8/8 (100%) | 38/39(97%) | 25/25(100%) | 1.000 | 1.000 |
| 2a (ⅰ) | Intended use and user in clinical pathway in introduction | 2/2 (100%) | 7/8 (88%) | 29/39(74%) | 21/25(84%) | 0.668 | 1.000 |
| 4a (ⅰ) | Participant-level eligibility criteria | 2/2 (100%) | 7/8 (88%) | 34/39(87%) | 23/25(92%) | 0.900 | 1.000 |
| 4a (ⅱ) | Input-data-level eligibility criteria | 0/2 (0%) | 0/7 (0%) | 2/39 (5%) | 1/25 (4%) | 1.000 | 1.000 |
| 4b | Integration into the trial setting | 2/2 (100%) | 1/8 (12%) | 12/39(31%) | 6/25 (24%) | 0.085 | 0.904 |
| 5 (ⅰ) | Version of the AI algorithm | 0/2 (0%) | 1/8 (12%) | 10/39(26%) | 6/25 (24%) | 0.823 | 1.000 |
| 5 (ⅱ) | Input-data acquisition and selection | 0/2 (0%) | 0/8 (0%) | 8/39 (21%) | 1/25 (4%) | 0.140 | 0.904 |
| 5 (ⅲ) | Handling of poor-quality or missing data | 0/2 (0%) | 0/8 (0%) | 0/38 (0%) | 0/24 (0%) | – | – |
| 5 (ⅳ) | Human-AI interaction and user expertise | 1/2 (50%) | 3/8 (38%) | 9/39 (23%) | 12/25(48%) | 0.218 | 0.904 |
| 5 (ⅴ) | Output of the AI intervention | 2/2 (100%) | 7/8 (88%) | 37/39(95%) | 24/25(96%) | 0.807 | 1.000 |
| 5 (ⅵ) | Contribution of output to decision-making | 0/2 (0%) | 6/8 (75%) | 17/34(50%) | 12/24(50%) | 0.290 | 0.904 |
| 19 | Performance errors and error analysis | 1/2 (50%) | 3/8 (38%) | 12/39(31%) | 11/25(44%) | 0.747 | 1.000 |
| 25 | Access to the AI intervention or code | 1/2 (50%) | 3/8 (38%) | 16/39(41%) | 11/25(44%) | 1.000 | 1.000 |

Table S10. Subgroup analysis (Intended Use)

| No | CONSORT-AI checklist | Info  &Education  n/33 (%) | Mental health  n/14 (%) | CDS^a^  n/14 (%) | Admin&Doc  n/12 (%) | etc^b^ 1/1(%) | *p-*value | *q*-value |
| --- | --- | --- | --- | --- | --- | --- | --- | --- |
| 1a, b (ⅰ) | AI intervention stated in title/abstract | 32/33 (97%) | 7/14 (50%) | 14/14 (100%) | 10/12 (83%) | 1/1 (100%) | 0.000 | 0.001 |
| 1a, b (ⅱ) | Intended use stated in title/abstract | 33/33 (100%) | 13/14 (93%) | 14/14 (100%) | 12/12 (100%) | 1/1 (100%) | 0.548 | 0.712 |
| 2a (ⅰ) | Intended use and user in clinical pathway in introduction | 22/33 (67%) | 13/14 (93%) | 13/14 (93%) | 11/12 (92%) | 0/1 (0%) | 0.041 | 0.133 |
| 4a (ⅰ) | Participant-level eligibility criteria | 30/33 (91%) | 14/14 (100%) | 12/14 (86%) | 9/12 (75%) | 1/1 (100%) | 0.194 | 0.360 |
| 4a (ⅱ) | Input-data-level eligibility criteria | 1/33 (3%) | 1/14 (7%) | 1/14 (7%) | 0/11 (0%) | 0/1 (0%) | 1.000 | 1.000 |
| 4b | Integration into the trial setting | 8/33 (24%) | 7/14 (50%) | 2/14 (14%) | 3/12(25%) | 1/1 (100%) | 0.170 | 0.360 |
| 5 (ⅰ) | Version of the AI algorithm | 4/33 (12%) | 5/14 (36%) | 3/14 (21%) | 4/12 (33%) | 1/1 (100%) | 0.251 | 0.408 |
| 5 (ⅱ) | Input-data acquisition and selection | 3/33 (9%) | 1/14 (7%) | 3/14 (21%) | 2/12 (17%) | 0/1 (0%) | 0.627 | 0.715 |
| 5 (ⅲ) | Handling of poor-quality or missing data | 0/31 (0%) | 0/14 (0%) | 0/14 (0%) | 0/12 (0%) | 0/1 (0%) | – | – |
| 5 (ⅳ) | Human-AI interaction and user expertise | 10/33 (30%) | 1/14 (7%) | 6/14 (43%) | 7/12 (58%) | 1/1 (100%) | 0.038 | 0.133 |
| 5 (ⅴ) | Output of the AI intervention | 32/33 (97%) | 14/14 (100%) | 12/14 (86%) | 11/12 (92%) | 1/1 (100%) | 0.393 | 0.567 |
| 5 (ⅵ) | Contribution of output to decision-making | 9/28 (32%) | 1/14 (7%) | 13/14 (93%) | 11/11 (100%) | 1/1 (100%) | <0.00001 | <0.00001 |
| 19 | Performance errors and error analysis | 12/33 (36%) | 4/14 (29%) | 4/14 (29%) | 6/12 (50%) | 1/1 (100%) | 0.660 | 0.715 |
| 25 | Access to the AI intervention or code | 9/33 (27%) | 5/14 (36%) | 8/14 (57%) | 8/12 (67%) | 1/1 (100%) | 0.056 | 0.147 |

^a^CDS: Clinical Decision Support.
^b^Not included in the exact test

Table S11. Subgroup analysis (Intended User)

| No | CONSORT-AI checklist | Clinician  n/23 (%) | Laypeople  n/38 (%) | Not reported  n/10 (%) | Others^a^  n/3 (%) | *p-*value | *q*-value |
| --- | --- | --- | --- | --- | --- | --- | --- |
| 1a, b (ⅰ) | AI intervention stated in title/abstract | 20/23 (87%) | 31/38 (82%) | 10/10 (100%) | 3/3 (100%) | 0.404 | 0.583 |
| 1a, b (ⅱ) | Intended use stated in title/abstract | 23/23 (100%) | 37/38 (97%) | 10/10 (100%) | 3/3 (100%) | 1.000 | 1.000 |
| 2a (ⅰ) | Intended use and user in clinical pathway in introduction | 21/23 (91%) | 36/38 (95%) | 0/10 (0%) | 2/3 (67%) | <0.00001 | <0.00001 |
| 4a (ⅰ) | Participant-level eligibility criteria | 20/23 (87%) | 36/38 (95%) | 7/10 (70%) | 3/3 (100%) | 0.057 | 0.226 |
| 4a (ⅱ) | Input-data-level eligibility criteria | 0/22 (0%) | 1/38 (3%) | 1/10 (10%) | 1/3 (33%) | 0.363 | 0.583 |
| 4b | Integration into the trial setting | 6/23 (26%) | 13/38 (34%) | 2/10 (20%) | 0/3 (0%) | 0.630 | 0.745 |
| 5 (ⅰ) | Version of the AI algorithm | 6/23 (26%) | 8/38 (21%) | 3/10 (30%) | 0/3 (0%) | 0.796 | 0.862 |
| 5 (ⅱ) | Input-data acquisition and selection | 4/23 (17%) | 1/38 (3%) | 2/10 (20%) | 2/3 (67%) | 0.106 | 0.230 |
| 5 (ⅲ) | Handling of poor-quality or missing data | 0/23 (0%) | 0/37 (0%) | 0/9 (0%) | 0/3 (0%) | – | – |
| 5 (ⅳ) | Human-AI interaction and user expertise | 10/23 (43%) | 12/38 (32%) | 1/10 (10%) | 2/3 (67%) | 0.178 | 0.330 |
| 5 (ⅴ) | Output of the AI intervention | 20/23 (87%) | 38/38 (100%) | 9/10 (90%) | 3/3 (100%) | 0.079 | 0.226 |
| 5 (ⅵ) | Contribution of output to decision-making | 23/23 (100%) | 6/38 (16%) | 3/4 (75%) | 3/3 (100%) | <0.00001 | <0.00001 |
| 19 | Performance errors and error analysis | 8/23 (35%) | 12/38 (32%) | 7/10 (70%) | 0/3 (0%) | 0.087 | 0.226 |
| 25 | Access to the AI intervention or code | 11/23 (48%) | 13/38 (34%) | 5/10 (50%) | 2/3 (67%) | 0.557 | 0.724 |

^a^Not included in the exact test

Table S12. Subgroup analysis (AI model family)

| No | CONSORT-AI checklist | GPT family n/45 (%) | Non-GPT n/22 (%) | Multiple models n/5 (%) | Not reported^a^ n/2 (%) | *p-*value | *q*-value |
| --- | --- | --- | --- | --- | --- | --- | --- |
| 1a, b (ⅰ) | AI intervention stated in title/abstract | 39/45 (87%) | 19/22 (86%) | 5/5 (100%) | 1/2 (50%) | 0.779 | 1.000 |
| 1a, b (ⅱ) | Intended use stated in title/abstract | 44/45 (98%) | 22/22 (100%) | 5/5 (100%) | 2/2 (100%) | 1.000 | 1.000 |
| 2a (ⅰ) | Intended use and user in clinical pathway in introduction | 34/45 (76%) | 18/22 (82%) | 5/5 (100%) | 2/2 (100%) | 0.483 | 1.000 |
| 4a (ⅰ) | Participant-level eligibility criteria | 39/45 (87%) | 20/22 (91%) | 5/5 (100%) | 2/2 (100%) | 0.629 | 1.000 |
| 4a (ⅱ) | Input-data-level eligibility criteria | 2/44 (5%) | 1/22 (5%) | 0/5 (0%) | 0/2 (0%) | 1.000 | 1.000 |
| 4b | Integration into the trial setting | 9/45 (20%) | 11/22 (50%) | 0/5 (0%) | 1/2 (50%) | 0.013 | 0.173 |
| 5 (ⅰ) | Version of the AI algorithm | 11/45 (24%) | 5/22 (23%) | 1/5 (20%) | 0/2 (0%) | 1.000 | 1.000 |
| 5 (ⅱ) | Input-data acquisition and selection | 7/45 (16%) | 2/22 (9%) | 0/5 (0%) | 0/2 (0%) | 0.632 | 1.000 |
| 5 (ⅲ) | Handling of poor-quality or missing data | 0/43 (0%) | 0/22 (0%) | 0/5 (0%) | 0/2 (0%) | – | – |
| 5 (ⅳ) | Human-AI interaction and user expertise | 13/45 (29%) | 10/22 (45%) | 1/5 (20%) | 1/2 (50%) | 0.376 | 1.000 |
| 5 (ⅴ) | Output of the AI intervention | 42/45 (93%) | 21/22 (95%) | 5/5 (100%) | 2/2 (100%) | 1.000 | 1.000 |
| 5 (ⅵ) | Contribution of output to decision-making | 23/40 (57%) | 11/21 (52%) | 0/5 (0%) | 1/2 (50%) | 0.054 | 0.352 |
| 19 | Performance errors and error analysis | 19/45 (42%) | 7/22 (32%) | 1/5 (20%) | 0/2 (0%) | 0.542 | 1.000 |
| 25 | Access to the AI intervention or code | 17/45 (38%) | 12/22 (55%) | 2/5 (40%) | 0/2 (0%) | 0.438 | 1.000 |

^a^Not included in the exact test

Table S13. Subgroup analysis (AI deployment)

| No | CONSORT-AI checklist | Off-the-shelf n/37 (%) | Customized n/32 (%) | Bespoke n/3 (%) | Not reported^a^ n/2 (%) | *p-*value | *q*-value |
| --- | --- | --- | --- | --- | --- | --- | --- |
| 1a, b (ⅰ) | AI intervention stated in title/abstract | 36/37 (97%) | 25/32 (78%) | 2/3 (67%) | 1/2 (50%) | 0.052 | 0.135 |
| 1a, b (ⅱ) | Intended use stated in title/abstract | 37/37 (100%) | 31/32 (97%) | 3/3 (100%) | 2/2 (100%) | 0.486 | 0.729 |
| 2a (ⅰ) | Intended use and user in clinical pathway in introduction | 24/37 (65%) | 31/32 (97%) | 2/3 (67%) | 2/2 (100%) | 0.009 | 0.060 |
| 4a (ⅰ) | Participant-level eligibility criteria | 33/37 (89%) | 28/32 (88%) | 3/3 (100%) | 2/2 (100%) | 1.000 | 1.000 |
| 4a (ⅱ) | Input-data-level eligibility criteria | 1/36 (3%) | 2/32 (6%) | 0/3 (0%) | 0/2 (0%) | 0.647 | 0.841 |
| 4b | Integration into the trial setting | 5/37 (14%) | 12/32 (38%) | 3/3 (100%) | 1/2 (50%) | 0.001 | 0.012 |
| 5 (ⅰ) | Version of the AI algorithm | 7/37 (19%) | 9/32 (28%) | 1/3 (33%) | 0/2 (0%) | 0.748 | 0.884 |
| 5 (ⅱ) | Input-data acquisition and selection | 5/37 (14%) | 4/32 (12%) | 0/3 (0%) | 0/2 (0%) | 1.000 | 1.000 |
| 5 (ⅲ) | Handling of poor-quality or missing data | 0/36 (0%) | 0/31 (0%) | 0/3 (0%) | 0/2 (0%) | – | – |
| 5 (ⅳ) | Human-AI interaction and user expertise | 10/37 (27%) | 12/32 (38%) | 2/3 (67%) | 1/2 (50%) | 0.318 | 0.591 |
| 5 (ⅴ) | Output of the AI intervention | 33/37 (89%) | 32/32 (100%) | 3/3 (100%) | 2/2 (100%) | 0.259 | 0.561 |
| 5 (ⅵ) | Contribution of output to decision-making | 21/31 (68%) | 12/32 (38%) | 1/3 (33%) | 1/2 (50%) | 0.032 | 0.105 |
| 19 | Performance errors and error analysis | 15/37 (41%) | 10/32 (31%) | 2/3 (67%) | 0/2 (0%) | 0.505 | 0.729 |
| 25 | Access to the AI intervention or code | 12/37 (32%) | 16/32 (50%) | 3/3 (100%) | 0/2 (0%) | 0.028 | 0.105 |

^a^Not included in the exact test

Table S14. Subgroup analysis (Clinical vignette)

| No | CONSORT-AI checklist | Vignette n/17 (%) | Non-vignette n/57 (%) | *p-*value | *q*-value |
| --- | --- | --- | --- | --- | --- |
| 1a, b (ⅰ) | AI intervention stated in title/abstract | 17/17 (100%) | 47/57 (82%) | 0.104 | 0.408 |
| 1a, b (ⅱ) | Intended use stated in title/abstract | 17/17 (100%) | 56/57 (98%) | 1.000 | 1.000 |
| 2a (ⅰ) | Intended use and user in clinical pathway in introduction | 13/17 (76%) | 46/57 (81%) | 0.736 | 1.000 |
| 4a (ⅰ) | Participant-level eligibility criteria | 10/17 (59%) | 56/57 (98%) | 0.000 | 0.001 |
| 4a (ⅱ) | Input-data-level eligibility criteria | 0/17 (0%) | 3/56 (5%) | 1.000 | 1.000 |
| 4b | Integration into the trial setting | 2/17 (12%) | 19/57 (33%) | 0.126 | 0.408 |
| 5 (ⅰ) | Version of the AI algorithm | 3/17 (18%) | 14/57 (25%) | 0.746 | 1.000 |
| 5 (ⅱ) | Input-data acquisition and selection | 2/17 (12%) | 7/57 (12%) | 1.000 | 1.000 |
| 5 (ⅲ) | Handling of poor-quality or missing data | 0/16 (0%) | 0/56 (0%) | – | – |
| 5 (ⅳ) | Human-AI interaction and user expertise | 6/17 (35%) | 19/57 (33%) | 1.000 | 1.000 |
| 5 (ⅴ) | Output of the AI intervention | 15/17 (88%) | 55/57 (96%) | 0.224 | 0.583 |
| 5 (ⅵ) | Contribution of output to decision-making | 11/14 (79%) | 24/54 (44%) | 0.035 | 0.225 |
| 19 | Performance errors and error analysis | 7/17 (41%) | 20/57 (35%) | 0.775 | 1.000 |
| 25 | Access to the AI intervention or code | 7/17 (41%) | 24/57 (42%) | 1.000 | 1.000 |

Table S15. Subgroup analysis (System prompt)

| No | CONSORT-AI checklist | System prompt n/20 (%) | No system prompt n/54 (%) | *p-*value | *q*-value |
| --- | --- | --- | --- | --- | --- |
| 1a, b (ⅰ) | AI intervention stated in title/abstract | 17/20 (85%) | 47/54 (87%) | 1.000 | 1.000 |
| 1a, b (ⅱ) | Intended use stated in title/abstract | 19/20 (95%) | 54/54 (100%) | 0.270 | 0.586 |
| 2a (ⅰ) | Intended use and user in clinical pathway in introduction | 17/20 (85%) | 42/54 (78%) | 0.746 | 0.969 |
| 4a (ⅰ) | Participant-level eligibility criteria | 18/20 (90%) | 48/54 (89%) | 1.000 | 1.000 |
| 4a (ⅱ) | Input-data-level eligibility criteria | 3/20 (15%) | 0/53 (0%) | 0.018 | 0.079 |
| 4b | Integration into the trial setting | 3/20 (15%) | 18/54 (33%) | 0.153 | 0.499 |
| 5 (ⅰ) | Version of the AI algorithm | 7/20 (35%) | 10/54 (19%) | 0.211 | 0.550 |
| 5 (ⅱ) | Input-data acquisition and selection | 7/20 (35%) | 2/54 (4%) | 0.001 | 0.014 |
| 5 (ⅲ) | Handling of poor-quality or missing data | 0/20 (0%) | 0/52 (0%) | – | – |
| 5 (ⅳ) | Human-AI interaction and user expertise | 7/20 (35%) | 18/54 (33%) | 1.000 | 1.000 |
| 5 (ⅴ) | Output of the AI intervention | 20/20 (100%) | 50/54 (93%) | 0.569 | 0.858 |
| 5 (ⅵ) | Contribution of output to decision-making | 11/19 (58%) | 24/49 (49%) | 0.594 | 0.858 |
| 19 | Performance errors and error analysis | 9/20 (45%) | 18/54 (33%) | 0.419 | 0.779 |
| 25 | Access to the AI intervention or code | 13/20 (65%) | 18/54 (33%) | 0.018 | 0.079 |

Table S16. Subgroup analysis (CONSORT-AI declared)

| No | CONSORT-AI checklist | CONSORT-AI declared n/9 (%) | Not declared n/65 (%) | *p-*value | *q*-value |
| --- | --- | --- | --- | --- | --- |
| 1a, b (ⅰ) | AI intervention stated in title/abstract | 8/9 (89%) | 56/65 (86%) | 1.000 | 1.000 |
| 1a, b (ⅱ) | Intended use stated in title/abstract | 9/9 (100%) | 64/65 (98%) | 1.000 | 1.000 |
| 2a (ⅰ) | Intended use and user in clinical pathway in introduction | 7/9 (78%) | 52/65 (80%) | 1.000 | 1.000 |
| 4a (ⅰ) | Participant-level eligibility criteria | 9/9 (100%) | 57/65 (88%) | 0.584 | 0.949 |
| 4a (ⅱ) | Input-data-level eligibility criteria | 1/9 (11%) | 2/64 (3%) | 0.330 | 0.613 |
| 4b | Integration into the trial setting | 3/9 (33%) | 18/65 (28%) | 0.707 | 1.000 |
| 5 (ⅰ) | Version of the AI algorithm | 4/9 (44%) | 13/65 (20%) | 0.197 | 0.613 |
| 5 (ⅱ) | Input-data acquisition and selection | 2/9 (22%) | 7/65 (11%) | 0.300 | 0.613 |
| 5 (ⅲ) | Handling of poor-quality or missing data | 0/8 (0%) | 0/64 (0%) | – | – |
| 5 (ⅳ) | Human-AI interaction and user expertise | 7/9 (78%) | 18/65 (28%) | 0.006 | 0.036 |
| 5 (ⅴ) | Output of the AI intervention | 9/9 (100%) | 61/65 (94%) | 1.000 | 1.000 |
| 5 (ⅵ) | Contribution of output to decision-making | 8/9 (89%) | 27/59 (46%) | 0.028 | 0.121 |
| 19 | Performance errors and error analysis | 5/9 (56%) | 22/65 (34%) | 0.273 | 0.613 |
| 25 | Access to the AI intervention or code | 8/9 (89%) | 23/65 (35%) | 0.003 | 0.036 |
