# Supplementary material for: Reporting Gaps and Challenges in Randomized Trials of Generative AI for Medical Use: Insights From a Systematic Review
Source: J Med Internet Res. 2026 Oct 28;28:e94543. doi: 10.2196/94543 (PMC13718382; doi:10.2196/94543)
Supplement: Supplementary file 10 [file jmir-v28-e94543-s008.docx]

**Table S17. Gen AI–specific reporting challenges mapped to CONSORT-AI checklist items**

| Challenge | CONSORT-AI item(s) | |
| --- | --- | --- |
| User dependency | 5(iv) | Human-AI interaction and user expertise |
|  | 5(vi) | Contribution of output to decision-making |
| Input variability | 4a(ii) | Input-data-level eligibility criteria |
|  | 5(ii) | Input-data acquisition and selection |
|  | 5(iii) | Handling of poor-quality or missing data |
| Model versioning and drift | 5(i) | Version of the AI algorithm |
| Handling of identifiable information | 5(iii) | Handling of poor-quality or missing data |
| System prompt | None | - |
| Inference settings | None | - |
| Date of model access or execution | None | - |
| Method of model access | None | - |
